# Synthesis and Structures of Ruthenium Carbonyl Complexes Bearing Pyridine-Alkoxide Ligands and Their Catalytic Activity in Alcohol Oxidation

**DOI:** 10.3389/fchem.2019.00394

**Published:** 2019-06-04

**Authors:** Xinlong Yan, Xiaohui Yue, Kang Liu, Zhiqiang Hao, Zhangang Han, Jin Lin

**Affiliations:** Hebei Key Laboratory of Organic Functional Molecules, College of Chemistry and Material Science, Hebei Normal University, Shijiazhuang, China

**Keywords:** ruthenium carbonyl complexes, alcohols oxidation, *t*-butyl hydroperoxide, pyridine alcohols, chemoselectivity

## Abstract

Reaction of Ru_3_(CO)_12_ with two equiv of 6-bromopyridine alcohols 6-bromopyCHROH [(R = C_6_H_5_ (**L1**); R = 4-CH_3_C_6_H_4_ (**L2**); R = 4-OMeC_6_H_4_ (**L3**); R = 4-ClC_6_H_4_ (**L4**); (R = 4-CF_3_C_6_H_4_ (**L5**); R = 2-OMeC_6_H_4_ (**L6**); R = 2-CF_3_C_6_H_4_ (**L7**)] and 6-bromopyC(Me)_2_OH (**L8**) in refluxing xylene afforded novel trinuclear ruthenium complexes [6-bromopyCHRO]_2_Ru_3_(CO)_8_ (**1a-1g**) and [6-bromopyC(Me)_2_O]_2_Ru_3_(CO)_8_ (**1h**). These complexes were characterized by FT-IR and NMR spectroscopy as well as elemental analysis. The structures of all the complexes were further confirmed by X-ray crystallographic analysis. In the presence of *tert*-butyl hydroperoxide (TBHP) as the source of oxidant, complexes **1a**-**1h** displayed high catalytic activities for oxidation of primary and secondary alcohols and most of oxidation reactions could be completed within 1 h at room temperature.

## Introduction

As a class of common starting materials, alcohols can beeasily converted into a variety of useful compounds via organic synthesis methods (Salvatore et al., 2001; Crabtree, 2017). Among all transfer strategies, oxidation of alcohols into their corresponding carbonyl compounds is one of the fundamental and important chemical reactions (Sheldon et al., 2000, 2002; Mallat and Baiker, 2004; Vazylyev et al., 2005; Parmeggiani and Cardona, 2012; Cao et al., 2014; Wang et al., 2017) and the oxidation products, including aldehydes, ketones and carboxylic acids, are important building blocks for synthesis of pharmaceuticals and fine chemicals (Caron et al., 2006; Bianchini and Shen, 2009; Simon and Li, 2012; Balaraman et al., 2013). Conventional oxidation methods to access these compounds usually require stoichiometric amounts of inorganic oxidants, such as chromium(VI) compounds (Canielli and Cardillo, 1984; Tojo and Fernández, 2007), hypervalent iodine reagents (Uyanik and Ishihara, 2009) or radical oxidants i.e., *N*-methylmorpholine-*N*-oxide (NMO) (Kumar et al., 2007; Gunasekaran et al., 2011; Saleem et al., 2013), 2,2,6,6-tetramethyl-1-piperidinyloxyl (TEMPO) (Dijksman et al., 2001; Wang et al., 2008; Allen et al., 2013). Such reactions often result in the generation of numerous wastes which caused serious environment problems. To ease this issue, great efforts have been devoted to the development of atom-economic and green methods. Molecular oxygen is one of the green oxidants and the H_2_O is the only by-product (Punniyamurthy et al., 2005). But in most aerobic alcohol oxidation systems, the additives e.g., TEMPO and large amounts of base are also needed, which makes the reaction system more complicated (Wang et al., 2005; Kumpulainen and Koskinen, 2009; Hoover et al., 2012). In addition, H_2_O_2_ is also used as an environmentally benign oxidant (Campestrini et al., 2004; Zhou et al., 2013; Ren et al., 2015; Vermaak et al., 2018). However, due to its limited oxidation capacity, the catalytic system should be assisted by carboxylic acid or H_2_SO_4_ as an additive to achieve a high efficiency (Dai et al., 2015; Miao et al., 2017). Compared with above oxidants, *tert*-butyl hydroperoxide (TBHP) is an alternative suitable oxidant and widely used in oxidation reactions, particularly in olefin epoxidation (Chen and Luck, 2016; Kashani et al., 2018) and C–H bond oxidation (Murahashi et al., 2000; Kudrik and Sorokin, 2017; Sarma et al., 2018). There are also some successful examples of using TBHP as an external oxidant in alcohol oxidation (Sarkar et al., 2014; Annunziata et al., 2018; Borah et al., 2018). In spite of this progress, less attention has been paid to this research area. Therefore, it is in an urgent demand to develop mild and efficient oxidation systems using TBHP as an oxidant.

It is well-known that transition metal complexes play a crucial role in oxidation process. Many transition metal catalysts including ruthenium (Shapley et al., 2000; Lybaert et al., 2017; Sarbajna et al., 2017; Moore et al., 2019), palladium (Stahl, 2004; Sigman and Jensen, 2006; Ho et al., 2018), copper (Velusamy et al., 2006; Jehdaramarn et al., 2018; Lagerspets et al., 2019), and iron (Coleman et al., 2010; Stanje et al., 2018) have been reported for promoting the oxidation of alcohols. Among them, ruthenium compounds are intensively studied because of their rich structures and various valence states. For example, Ramesh and co-workers reported that ruthenium(II) carbonyl 2-(arylazo)phenolate complexes could oxidize sensitive group-contained alcohols with moderate to high conversion (Kumar et al., 2007). The group of Zhang synthesized several ruthenium complexes containing 2-(biphenylazo)phenolate ligands that successfully achieved high catalytic activity in the presence of NMO without diminishing chemoselectivity (Tang et al., 2018). However, very few examples were focused on di-or tri-nuclear ruthenium complexes and their applications in organic reactions were limited. Recently, we have reported a series of triruthenium carbonyl complexes and their efficient oxidation behavior toward secondary alcohols, while these Ru compounds showed poor reactivity in oxidation of primary alcohols (Hao et al., 2018a,b, 2019). As part of our continuing efforts in developing novel ruthenium carbonyl complexes and their applications in alcohol oxidation, herein, we reported the synthesis and characterization of several ruthenium carbonyl complexes supported by pyridine-alkoxide ligands and their catalytic properties in the oxidation of primary and secondary alcohols using TBHP as an oxidant.

## Experimental

### Materials and Methods

All manipulations were performed under a nitrogen atmosphere using standard Schlenk techniques. Solvents for reaction were distilled from appropriate drying agents under N_2_ before use. All the chemical reagents were purchased from commercial sources. Ru_3_(CO)_12_ was prepared by literature methods (Fauré et al., 2003). NMR spectra were measured using a Bruker Avance III-500 NMR spectrometer at room temperature with TMS as internal standard. Melting points were determined using an SGW X-4A Digital Melting Point Apparatus. IR spectra were recorded as KBr disks on a Thermo Fisher iS 50 spectrometer in the range 4,000–600 cm^−1^ and elemental analyses were performed on a Vario EL III analyzer.

### Preparation of 6-bromopyCH(2-CF_3_C_6_H_4_)OH (L7)

To a dried Et_2_O (30 mL) of 2, 6-dibromopyridine (3.55 g, 15 mmol) at −78°C, *n*-BuLi (9.5 mL, 15 mmol) was added dropwise via a syringe in 10 min and the solution was stirred at −78°C for 1 h. After addition of 2-(trifluoromethy)benzaldehyde (2.61 g, 15 mmol), the mixture was allowed to warm to room temperature and stirred overnight. The reaction solution was neutralized with aqueous NH_4_Cl and the organic phase was separated. The aqueous layer was extracted with CH_2_Cl_2_ (3 × 10 mL), and combined organic fractions were dried over MgSO_4_ and the residue was placed in an Al_2_O_3_ column with ethyl acetate/petroleum ether as an eluent to give **L7** as a off-white powders (2.04 g, 46%). ^1^H NMR (CDCl_3_, 500 MHz, 298 K): δ 7.69 (d, *J* = 7.9 Hz, 1 H, Py-H), 7.53 (t, *J* = 7.7 Hz, 1 H, Py-H), 7.45–7.49 (m, 2 H, C_6_H_4_), 7.41 (d, *J* = 8.2 Hz, 2 H, C_6_H_4_), 6.96 (d, *J* = 7.7 Hz, 1 H, Py-H), 6.15 (s, 1 H, CH), 4.93 (s, 1 H, OH). ^13^C NMR (CDCl_3_, 125 MHz, 298 K): δ 161.9, 141.1, 140.8, 139.5, 132.6, 129.9, 128.4, 128.2, 127.2, 125.5 (q, *J*_C−F_ = 5.5 Hz), 123.1 (q, *J*_C−F_ = 272 Hz), 120.6, 69.5 ppm.

### Preparation of (6-bromopyCHC_6_H_5_O)_2_Ru_3_(CO)_8_ (1a)

A solution of ligand precursor **L1** (0.248 g, 0.938 mmol) and Ru_3_(CO)_12_ (0.300 g, 0.469 mmol) in 30 mL of xylene was refluxed for 10 h. After evaporation of the solvent in vacuo, the residue was placed in an Al_2_O_3_ column. Elution with ethyl acetate/petroleum ether gave **1a** as orange crystals (yield 0.307 g, 62%). Mp: 168–169°C. Anal. Calc. for C_32_H_18_Br_2_N_2_O_10_Ru_3_: C, 36.48; H, 1.72; N, 2.66. Found (%): C, 36.30; H, 1.85; N, 2.71. ^1^H NMR (CDCl_3_, 500 MHz, 298 K): δ 7.61 (d, *J* = 7.7 Hz, 2 H, Py-H), 7.25–7.29 (m, 8 H, Py-H, C_6_H_4_), 7.15 (d, *J* = 6.5 Hz, 4 H, C_6_H_4_), 6.62 (d, *J* = 7.7 Hz, 2 H, Py-H), 5.58 (s, 2 H, CH) ppm. ^13^C NMR (CDCl_3_, 125 MHz, 298 K): δ 205.9, 202.9, 200.8, 190.1, 170.8, 144.8, 143.4, 138.2, 128.7, 128.4, 128.3, 128.0, 120.0, 91.7 ppm. IR (υ_CO_, KBr, cm^−1^): 2081(s), 2015(s), 1998(vs), 1909(s).

### Preparation of [6-bromopyCH(4-MeC_6_H_4_O)]_2_Ru_3_(CO)_8_ (1b)

Complex **1b** was prepared in a similar procedure to that described above for preparation of **1a**. Reaction of **L2** (0.261 g, 0.938 mmol) with Ru_3_(CO)_12_ (0.300 g, 0.469 mmol) in 30 mL of xylene generated complex **1b** as orange crystals (yield 0.358 g, 70%). Mp: 173–175°C. Anal. Calc. for C_34_H_22_Br_2_N_2_O_10_Ru_3_: C, 37.76; H, 2.05 N, 2.59. Found (%): C, 37.91; H, 2.19, N, 2.45. ^1^H NMR (CDCl_3_, 500 MHz, 298 K): δ 7.60 (d, *J* = 7.7 Hz, 2 H, Py-H), 7.26 (t, *J* = 7.7 Hz, 2 H, Py-H), 7.09 (d, *J* = 7.9 Hz, 4 H, C_6_H_4_), 7.04 (d, *J* = 8.0 Hz, 4 H, C_6_H_4_), 6.60 (d, *J* = 7.7 Hz, 2 H, Py-H), 5.54 (s, 2 H, CH), 2.30 (s, 6 H, CH_3_) ppm. ^13^C NMR (CDCl_3_, 125 MHz, 298 K): δ 206.0, 203.0, 200.9, 190.2, 171.0, 144.7, 140.5, 138.1, 138.0, 129.3, 128.2, 127.9, 119.9, 91.5, 21.4 ppm. IR (υ_CO_, KBr, cm^−1^): 2085(s), 2015(s), 2000(s), 1909(s).

### Preparation of [6-bromopyCH(4-OMeC_6_H_4_O)]_2_Ru_3_(CO)_8_ (1c)

Complex **1c** was prepared in a similar procedure to that described above for preparation of **1a**. Reaction of **L3** (0.276 g, 0.938 mmol) with Ru_3_(CO)_12_ (0.300 g, 0.469 mmol) in 30 ml of xylene generated complex **1c** as orange crystals (yield 0.337 g, 65%). Mp: 176–177°C. Anal. Calc. for C_34_H_22_Br_2_N_2_O_12_Ru_3_: C, 36.67; H, 1.99, N, 2.52. Found (%): C, 36.53; H, 2.10, N, 2.44. ^1^H NMR (CDCl_3_, 500 MHz, 298 K): δ 7.59 (d, *J* = 7.7 Hz, 2 H, Py-H), 7.26 (t, *J* = 7.7 Hz, 2 H, Py-H), 7.07 (d, *J* = 8.6 Hz, 4 H, C_6_H_4_), 6.81 (d, *J* = 8.6 Hz, 4 H, C_6_H_4_), 6.60 (d, *J* = 7.7 Hz, 2 H, Py-H), 5.55 (s, 2 H, CH), 3.76 (s, 6H, OCH_3_) ppm. ^13^C NMR (CDCl_3_, 125 MHz, 298 K): δ 206.0, 203.0, 200.9, 190.2 171.1, 159.6, 144.7, 138.1, 135.9, 129.3, 128.2, 120.0, 114.1, 91.2, 55.4 ppm. IR (υ_CO_, KBr, cm^−1^): 2078(s), 2004(s), 1993(vs), 1912(s).

### Preparation of [6-bromopyCH(4-ClC_6_H_4_O)]_2_Ru_3_(CO)_8_ (1d)

Complex **1d** was prepared in a similar procedure to that described above for preparation of **1a**. Reaction of **L4** (0.281 g, 0.938 mmol) with Ru_3_(CO)_12_ (0.300 g, 0.469 mmol) in 30 mL of xylene generated complex **1d** as orange crystals (yield 0.267 g, 51%). Mp: 182–183°C. Anal. Calc. for C_32_H_16_Br_2_Cl_2_N_2_O_10_Ru_3_: C, 34.24; H, 1.44, N, 2.50. Found (%): C, 34.34; H, 1.52, N, 2.48. ^1^H NMR (CDCl_3_, 500 MHz, 298 K): δ 7.64 (d, *J* = 7.5 Hz, 2 H, Py-H), 7.31 (t, *J* = 7.7 Hz, 2 H, Py-H), 7.29 (d, *J* = 8.3 Hz, 4 H, C_6_H_4_), 7.08 (d, *J* = 8.3 Hz, 4 H, C_6_H_4_), 6.61 (d, *J* = 7.7 Hz, 2 H, Py-H), 5.54 (s, 2 H, CH) ppm. ^13^C NMR (CDCl_3_, 125 MHz, 298 K): δ 205.8, 202.8, 200.9,189.7, 170.1,144.9, 141.8, 138.3, 134.2, 129.3, 129.0, 128.6, 119.9, 90.9 ppm. IR (υ_CO_, KBr, cm^−1^): 2084(s), 2012(s), 1998(s), 1915(s).

### Preparation of [6-bromopyCH(4-CF_3_C_6_H_4_O)]_2_Ru_3_(CO)_8_ (1e)

Complex **1e** was prepared in a similar procedure to that described above for preparation of **1a**. Reaction of **L5** (0.339 g, 0.938 mmol) with Ru_3_(CO)_12_ (0.300 g, 0.469 mmol) in 30 mL of xylene generated complex **1e** as orange crystals (yield 0.267 g, 48%). Mp: 177–179°C. Anal. Calc. for C_34_H_16_Br_2_F_6_N_2_O_10_Ru_3_: C, 34.33; H, 1.36, N, 2.36. Found (%): C, 34.44; H, 1.30, N, 2.43. ^1^H NMR (CDCl_3_, 500 MHz, 298 K): δ 7.67 (d, *J* = 7.7 Hz, 2 H, Py-H), 7.57 (d, *J* = 8.1 Hz, 4 H, C_6_H_4_), 7.35 (t, *J* = 7.8 Hz, 2 H, Py-H), 7.29 (d, *J* = 8.0 Hz, 4 H, C_6_H_4_), 6.64 (d, *J* = 7.6 Hz, 2 H, Py-H), 5.61 (s, 2 H, CH) ppm. ^13^C NMR (CDCl_3_, 125 MHz, 298 K): δ 205.6, 202.7, 200.9, 189.5, 169.7, 146.9, 145.0, 138.5, 130.7, 128.9, 128.2, 125.8 (q, *J*_C−F_ = 2.7 Hz), 123.1 (q, *J*_C−F_ = 270.5 Hz), 120.0, 91.1 ppm. IR (υ_CO_, KBr, cm^−1^): 2083(s), 2017(s), 1993(s), 1912(s).

### Preparation of [6-bromopyCH(2-OMeC_6_H_4_O)]_2_Ru_3_(CO)_8_ (1f)

Complex **1f** was prepared in a similar procedure to that described above for preparation of **1a**. Reaction of **L6** (0.276 g, 0.938 mmol) with Ru_3_(CO)_12_ (0.300 g, 0.469 mmol) in 30 mL of xylene generated complex **1f** as orange crystals (yield 0.298 g, 57%). Mp: 183–184°C. Anal. Calc. for C_34_H_22_Br_2_N_2_O_12_Ru_3_: C, 36.67; H, 1.99, N, 2.52. Found (%): C, 36.75; H, 2.12, N, 2.45. ^1^H NMR (CDCl_3_, 500 MHz, 298 K): δ 7.59 (d, *J* = 7.7 Hz, 2 H, Py-H), 7.24–7.19 (m, 4 H, C_6_H_4_, Py-H), 6.85 (d, *J* = 8.2 Hz, 2 H, C_6_H_4_), 6.76 (t, *J* = 7.4 Hz, 2 H, C_6_H_4_), 6.61 (d, *J* = 7.6 Hz, 2 H, Py-H), 6.50 (d, *J* = 7.5 Hz, 2 H, C_6_H_4_), 6.04 (s, 2 H, CH), 3.94 (s, 6 H, OCH_3_) ppm. ^13^C NMR (CDCl_3_, 125 MHz, 298 K): δ 206.2, 203.1, 201.1, 190.7, 171.2, 157.3, 144.9, 138.0, 131.7, 129.4, 128.3, 128.1, 120.0, 119.2, 110.3, 84.9, 55.2 ppm. IR (υ_CO_, KBr, cm^−1^): 2086 (s), 2020 (vs), 1995 (s), 1960 (s), 1905 (s).

### Preparation of [6-bromopyCH(2-CF_3_C_6_H_4_O)]_2_Ru_3_(CO)_8_ (1g)

Complex **1g** was prepared in a similar procedure to that described above for preparation of **1a**. Reaction of **L7** (0.339 g, 0.938 mmol) with Ru_3_(CO)_12_ (0.300 g, 0.469 mmol) in 30 mL of xylene generated complex **1g** as orange crystals (yield 0.212 g, 38%). Mp: 186–188°C. Anal. Calc. for C_34_H_16_Br_2_F_6_N_2_O_10_Ru_3_: C, 34.33; H, 1.36, N, 2.36. Found (%): C, 34.25; H, 1.41, N, 2.43. ^1^H NMR (CDCl_3_, 500 MHz, 298 K): δ 7.69 (d, J = 7.8 Hz, 2 H, Py-H), 7.62 (d, *J* = 7.9 Hz, 2 H, C_6_H_4_), 7.43 (t, J = 7.6 Hz, 2 H, Py-H), 7.35–7.30 (m, 6 H, C_6_H_4_), 6.51 (d, *J* = 7.7 Hz, 2 H, Py-H), 5.61 (s, 2 H, CH) ppm. ^13^C NMR (CDCl_3_, 125 MHz, 298 K): δ 204.9, 203.1, 200.8, 190.0, 169.9, 145.3, 141.9, 138.5, 132.3, 129.9, 128.7, 127.9, 126.4, 125.7 (q, *J*_C−F_ = 4.6 Hz), 123.0 (q, *J*_C−F_ = 273.8 Hz), 119.8, 85.5 ppm. IR (υ_CO_, KBr, cm^−1^): 2089(m), 2022(s), 2001(s), 1969(s), 1914(s).

### Preparation of [6-bromopyCH(Me)_2_O]_2_Ru_3_(CO)_8_ (1h)

Complex **1h** was prepared in a similar procedure to that described above for preparation of **1a**. Reaction of **L8** (0.202 g, 0.938 mmol) with Ru_3_(CO)_12_ (0.300 g, 0.469 mmol) in 30 mL of xylene generated complex **1h** as orange crystals (yield 0.305 g, 68%). Mp: 171–172°C. Anal. Calc. for C_24_H_18_Br_2_N_2_O_10_Ru_3_: C, 30.11; H, 1.89; N, 2.93. Found (%): C, 30.25; H, 1.99; N, 2.81. ^1^H NMR (CDCl_3_, 500 MHz, 298 K): δ 7.51 (d, *J* = 7.6 Hz, 2 H, Py-H), 7.41 (t, *J* = 7.7 Hz, 2 H, Py-H), 6.89 (d, *J* = 7.6 Hz, 2 H, Py-H), 1.37 (s, 6 H, CH_3_), 1.18 (s, 6 H, CH_3_) ppm. ^13^C NMR (CDCl_3_, 125 MHz, 298 K) δ 206.9, 202.9, 202.7, 191.0, 175.7, 145.6, 138.8, 128.2, 118.7, 87.7, 34.7, 31.1 ppm. IR (υ_CO_, KBr, cm^−1^): 2080(s), 2007(s), 1966(s), 1906(s).

### General Procedure for Catalytic Oxidation of Alcohols

An alcohol substrate (1.0 mmol), complex **1c** (0.005 mmol) and TBHP (2.5 mmol) was placed in a 2-neck 25 mL round bottom flask and degassed 2 times. Two milliliter of dried CH_3_CN was then added and the resulting mixture was reacted at room temperature for 1 h under an N_2_ atmosphere. After the reaction was complete, the solvent was removed under reduced pressure and the residue was purified by Al_2_O_3_ column chromatography (eluent ethylacetate/petroleum ether v/v = 1/15) to afford the desired product, which was identified by comparison with the authentic sample through NMR and GC analyses.

### Crystal Structural Determination

Single crystals of complexes **1a-1h** suitable for X-ray crystal structural analysis were obtained from a CH_2_Cl_2_/*n*-hexane mixed solvent system. Data collection was performed on a Bruker SMART 1000 diffractometer, using graphite-monochromated Mo-K radiation (ω*-*φ scans, λ = 0.71073 Å). The structures were solved by direct methods and refined by full-matrix least squares. All calculations were using SHELXTL crystallographic software packages (Sheldrick, 1997). The crystal data and summary of X-ray data collection are presented in Tables S2, S3.

## Results and Discussion

### Synthesis of Ligands and Ruthenium Complexes

6-bromopyridine alcohol ligands **L1**-**L6 and L8** were synthesized according to the literature procedure (Tsukahara et al., 1997; Song and Morris, 2004) and identified by NMR and elemental analysis prior to use. **L7** was synthesized following similar methods. 2,6-Dibromopyridine reacted with *n*-BuLi and 1 equiv of *o*-substituted aldehydes was added to the reaction mixture, then hydrolysis led to the target ligand.

**Scheme 1:**
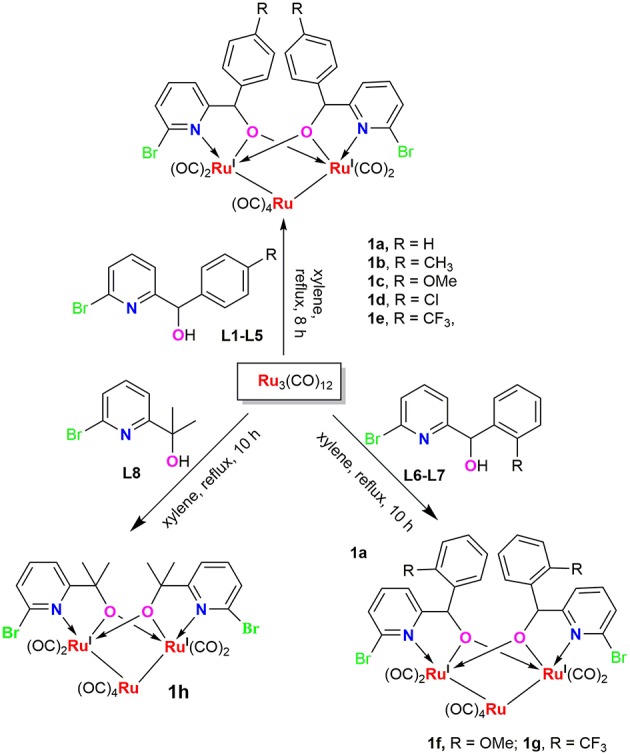
Synthetic routes of Ru complexes.

Ruthenium clusters were prepared in moderate to high yields from Ru_3_(CO)_12_ by treating with 2.0 equiv of ligands **L1**-**L8** in refluxing xylene, respectively. The general synthetic route for these new compounds is depicted in Scheme 1. These trinuclear Ru complexes were identified by FI-IR, NMR spectroscopy and elemental analysis. The FT-IR spectra of all the complexes exhibit several absorption peaks around 1906–1950 cm^−1^, which can be assigned to the characteristic stretching vibration of the terminally coordinated CO. In the ^1^H NMR spectra of **1a**-**1g**, the characteristic signal of –OH disappeared and the singlets resonance for the methyne adjacent to oxygen were observed at 5.5–6.1 ppm, which were shifted upfield when compared to those in the free ligands (L1-L7). In the ^13^C NMR spectra, the resonance signals around 85–92 ppm correspond to the methynes mentioned above, which are in good accordance with compound [pyC(Me)_2_O]_2_Ru_3_(CO)_8_ (δ 87.6 ppm) and [pyCHC_6_H_5_O]_2_Ru_3_(CO)_8_ (δ 89.3 ppm), which were previously reported in the literature (Hao et al., 2018b).

### Crystal Structures of Complexes 1a-1h

**Figure 1:**
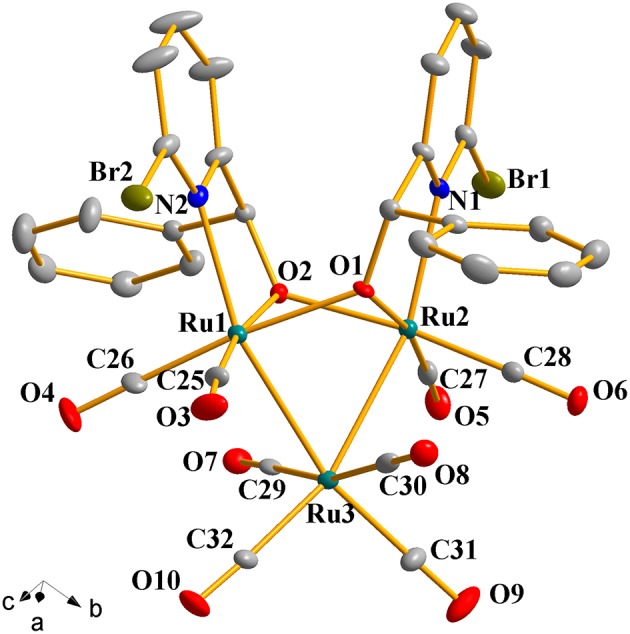
Perspective view of **1a** with thermal ellipsoids are drawn at the 30% probability level. Hydrogens have been omitted for clarity. The selected bond lengths (Å) and angles (°): Ru(1)-Ru(3) 2.7743(10), Ru(2)-Ru(3) 2.7625(10), Ru(1)-O(1) 2.140(5), Ru(2)-O(1) 2.084(6), Ru(1)-N(2) 2.237(7), Ru(2)-N(1) 2.252(7); N(2)-Ru(1)-Ru(3) 157.9(2), N(2)-Ru(1)-Ru(2) 102.2(2), Ru(2)-O(1)-Ru(1) 91.4(2), Ru(1)-O(2)-Ru(2) 91.1(2), Ru(2)-Ru(3)-Ru(1) 66.20(3).

**Figure 2:**
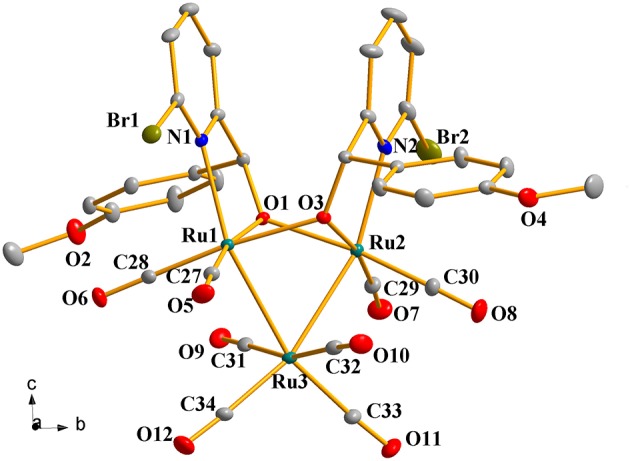
Perspective view of **1c** with thermal ellipsoids are drawn at the 30% probability level. Hydrogens and solvent have been omitted for clarity. The selected bond lengths (Å) and angles (°): Ru(1)-O(1) 2.083(3), Ru(2)-O(1) 2.132(3), Ru(1)-Ru(3) 2.7649(5), Ru(2)-Ru(3) 2.7552(6), Ru(1)-N(1) 2.258(4), Ru(2)-N(2) 2.240(4); Ru(1)-O(1)-Ru(2) 92.33(12), Ru(2)-O(3)-Ru(1) 92.00(12), N(1)-Ru(1)-Ru(3) 157.10(10), N(2)-Ru(2)-Ru(3) 157.01(12), Ru(2)-Ru(3)-Ru(1) 66.848(15).

**Figure 3:**
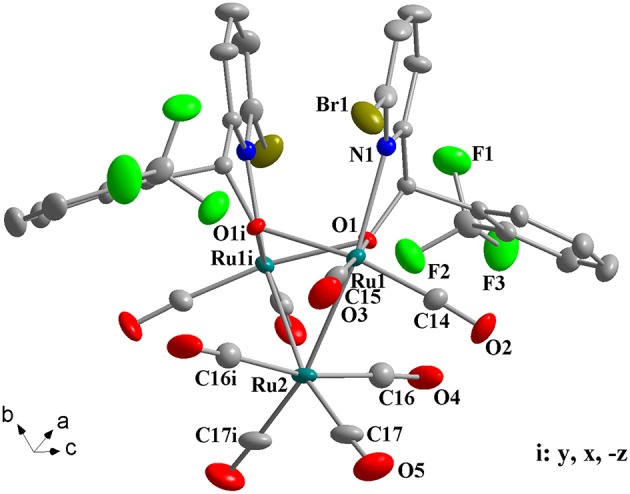
Perspective view of **1g** with thermal ellipsoids are drawn at the 30% probability level. Hydrogens have been omitted for clarity. The selected bond lengths (Å) and angles (°): Ru(1)-O(1) 2.089(4), Ru(1)-O(1i) 2.147(4), Ru(1)-N(1) 2.313(5), Ru(2)-Ru(1) 2.7560(7), Ru(2)-Ru(1i) 2.7560(7), Ru(1)-O(1)-Ru(1i) 92.06(15), N(1)-Ru(1)-Ru(1i) 97.72(13), Ru(1)-Ru(2)-Ru(1i) 67.17(2).

Complexes **1a**-**1h** were further characterized by X-ray crystallography. The molecular structures of **1a**, **1c**, and **1g** are shown in Figures 1–3 with selected bond lengths and angles, respectively. Those of **1b**, **1d**-**1f**, and **1h** are shown in Figures S1–S5, respectively. Details of the structural parameters are also given in Tables S2, S3. X-ray diffraction analysis shows that all the complexes **1a**-**1h** are trinuclear ruthenium clusters accompanied by two pyridylalkoxo ligands simultaneously via their pyridyl N and hydroxy O atoms. Three Ru atoms adopt a pseudooctahedral coordinated mode. The unit cell of **1a** contains two crystallographically-independent molecules which possess similar connectivity and only one molecular structure is depicted in Figure 1 for clarity. In complexes **1a**-**1h**, the distances between the two Ru atoms directly connected to the ligands are in the range of 3.0236(10)-3.0516(10) Å, which are comparable to the Ru–Ru bond distances in complexes (μ-OC_6_H_4_OMe-2)_2_Ru_3_(CO)_8_ (3.012(1) Å) (Santini et al., 1987) and [PyCH = C(Ph)O]_2_Ru_3_(CO)_8_ (3.0693(6) Å) (Ma et al., 2017). The bond lengths of Ru(1)–N(1) varying from 2.252(7) Å to 2.330(14) Å observed in all complexes are slightly longer than those Ru–N bond lengths found in complexes {μ_2_-μ^5^:η^1^-(C_5_H_4_N)(C_9_H_5_)}Ru_3_(CO)_9_ [2.164(3) Å] (Chen et al., 2010) and (6-bromopyCMeC_6_H_5_O)Ru_3_(CO)_9_ (2.229(4) Å) (Hao et al., 2018b). The Ru(1)–O(1) bond lengths for **1a**-**1h** are all similar and are in the range of 2.072(3)-2.093(5) Å, showing that the substitutions at 2- or 4- positions of benzene ring have no significant effect on bond lengths.

### Catalytic Activity on Oxidation of Alcohols

**Figure 4:**
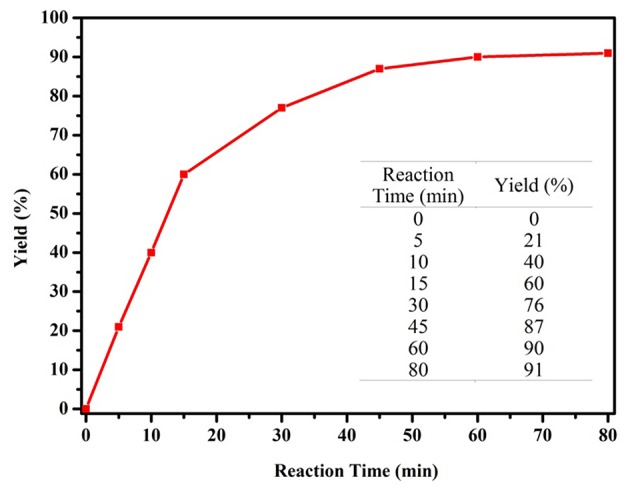
Influence of the reaction time on catalytic performance of complex **1a** (1-phenylethanol 1.0 mmol, complex **1a** 0.5 mol%, TBHP 2.5 mmol, CH_3_CN 2.0 mL).

We commenced our study by using complex **1a** as precatalyst and 1-phenylethanol as a simple substrate to obtain the appropriate conditions. Initially, various oxidants (NMO, H_2_O_2_, *t*-BuOOH, TEMPO) were tested to oxidize 1-phenylethanol. Among diffrerent oxidants applied in this studies, *t*-BuOOH was found to be the best oxidant and acetophenone can be obtained in a high yield of 75% (Table S1). The effect of solvent on this oxidation process was then evaluated (Table 1, entries 1–6). When the reaction was performed in CH_2_Cl_2_, THF or dioxide, the oxidation product was obtained in low yields (<55%). When using toluene or acetone as solvent, the the yield was enhanced slightly. To our delight, the yield of the desired ketone in CH_3_CN was 85%, which is higher than that in other solvents. Thus, acetonitrile was selected as the optimal solvent. The subsequent lowering the loading of **1a** from 2.0 to 0.5 mol% did not significantly affect the yield, thus only 0.5 mol% catalyst **1a** is sufficient for catalyzing the present reaction (Table 1, entries 3 and 7–9). Subsequently, the effect of TBHP on the reaction was examined. Upon increasing the amounts of TBHP from 1.0 to 3.0 mmol, the yield of acetophenone was gradually improved to 91%, and 2.5 mmol of TBHP was selected as the most suitable amount from the view of cost-saving (Table 1, entries 10–13). It was found that the reaction temperature had an obvious impact on the reaction efficiency. As shown in Table 1, when elevating the temperature from room temperature to 50 or 80°C, the yield of product decreased dramatically to 72 and 54%, respectively (Table 1, entries 14 and 15). Furthermore, the effect of reaction time on the rate of oxidation was investigated (Figure 4). When the reaction time was extended from 0 to 20 min, an almost linear increase of the yield was observed during the oxidation of 1-phenylethanol. After 40 min, the yield increased slightly, and further extension of the time after 60 min could hardly increase the yield. Finally, control experiments indicated that only traces (8%) of the desired product was formed without using complex **1a** and almost no oxidation product was generated in the absence of TBHP (Table 1, entries 16 and 17).

**Table 1:** Oxidation of 1-phenylethanol catalyzed by complex 1a under various conditions^a^.

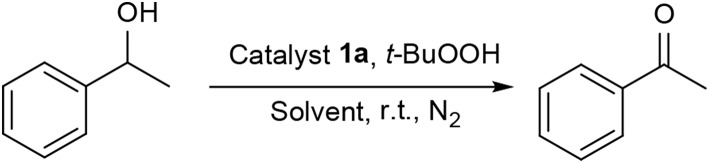					
**Entry**	**Cat. (mol%)**	**TBHP (mmol)**	**Solvent**	**Yield^b^(%)**	**TOF^c^(h^−1^)**
1	1.0	2.0	Toluene	64	113
2	1.0	2.0	Acetone	77	120
3	1.0	2.0	CH_3_CN	85	138
4	1.0	2.0	CH_2_Cl_2_	53	69
5	1.0	2.0	THF	44	60
6	1.0	2.0	Dioxide	40	31
7	2.0	2.0	CH_3_CN	88	90
8	1.5	2.0	CH_3_CN	86	102
9	0.5	2.0	CH_3_CN	85	228
10	0.5	1.0	CH_3_CN	80	129
11	0.5	1.5	CH_3_CN	84	164
12	0.5	2.5	CH_3_CN	90	240
13	0.5	3.0	CH_3_CN	91	257
14^d^	0.5	2.5	CH_3_CN	72	206
15^e^	0.5	2.5	CH_3_CN	54	72
16	–	2.5	CH_3_CN	Trace	–
17	0.5	–	CH_3_CN	–	–

a *Reaction conditions: 1-phenylethanol (1.0 mmol), solvent (2.0 mL), reaction time 1 h*.

b *Yield was determined by GC*.

c *TOF was calculated at 30% conversion of 1-phenylethanol*.

d *T = 50°C*.

e *T = 80°C*.

The above interesting results encouraged us to continue the optimization study using different ruthenium complexes and the obtained results are summaried in Table 2. The complexes containing electronically rich ligands (**1a-c**, **1f**, and **1h**) exhibited higher catalytic activity than those bearing electronically poor ligands (**1d**, **1e**, and **1g**), suggesting that the catalytic behavior of Ru complexes was influenced by the electronic nature of the ligands. Catalysts **1f** and **1g** with *ortho*-substituents in the phenyl ring exhibited lower catalytic activity than their *para*-substituted analogs **1c** and **1e** (Table 2, entries 3–4 vs. 6–7). This difference is likely due to the fact that substitutions at 2-position of ligands caused more steric hindrance around the metal centers, thus influencing the coordination of metals with substrates. Besides, catalytic oxidation of 1-phenylethanol was also carried out in the presence of Ru_3_(CO)_12_ and the yield of the desired product was only 30% (Table 2, entry 9). Thus, the optimized reaction conditions are as follows: alcohol (1.0 mmol), TBHP (2.5 mmol), catalyst **1c** (0.5 mol%), reaction time (1 h) at room temperature.

**Table 2:** Comparison of catalytic activity of Ru complexes^a^.

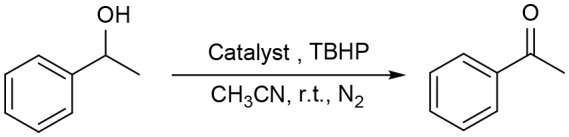					
**Entry**	**Catalyst**	**TBHP (mmol)**	**Solvent**	**Yield^b^(%)**	**TOF^c^(h^−1^)**
1	**1a**	2.5	CH_3_CN	91	240
2	**1b**	2.5	CH_3_CN	93	273
3	**1c**	2.5	CH_3_CN	96	281
4	**1d**	2.5	CH_3_CN	87	228
5	**1e**	2.5	CH_3_CN	85	220
6	**1f**	2.5	CH_3_CN	90	243
7	**1g**	2.5	CH_3_CN	74	210
8	**1h**	2.5	CH_3_CN	92	256
9	Ru_3_(CO)_12_	2.5	CH_3_CN	30	–

a *Reaction conditions: 1-phenylethanol (1.0 mmol), catalyst (0.5 mol%), solvent (2.0 mL), reaction time 1 h*.

b *Yield was determined by GC*.

c *TOF was calculated at 30% conversion of 1-phenylethanol*.

Under optimized conditions, we set out to test the catalytic performance of complex **1c** in oxidation of different secondary alcohols. As listed in Table 3, a diverse array of functional groups including methyl-, chloro-, and trifluoromethyl- etc. on the phenyl ring of substituted 1-phenylethanol were tolerated (Table 3, entries 1–7). Additionally, several sterically encumbered substrates undergo oxidation in >90% yields (Table 3, entries 8–10). The fused-ring alcohols, that are 1,2,3,4-tetrahydro-1-naphthol and 1-indanol could be also converted to target product in 93 and 92% yields, respectively (Table 3, entries 11 and 12). As for secondary aliphatic alcohols, a prolonged reaction time (3h) was required to achieve high yields (Table 3, entries 13–16).

**Table 3:** Oxidation of various of secondary alcohols to ketones catalyzed by 1c^a^.

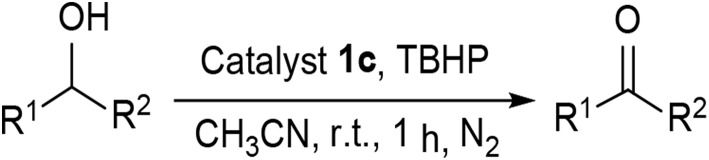				
**Entry**	**Alcohol**	**Conv.^b^ (%)**	**Yield^c^ (%)**	**TOF^d^ (h^−1^)**
	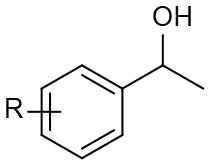			
1	R = 4-Me	96	92	286
2	R = 3-Me	99	93	290
3	R = 4-OMe	97	94	305
4	R = 4-Cl	96	93	270
5	R = 4-Br	94	90	256
6	R = 3-Br	91	85	250
7	R = 4-CF_3_	90	87	243
8	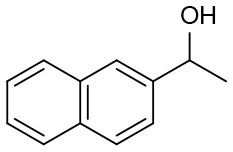	96	90	285
9	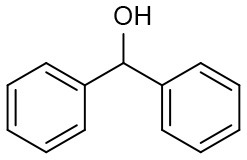	97	91	275
10	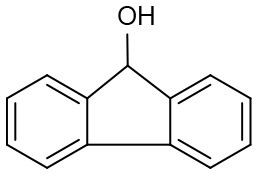	99	96	292
11	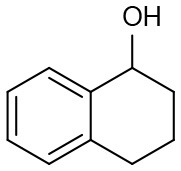	97	93	274
12	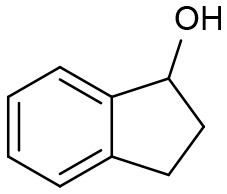	98	92	280
13^e^	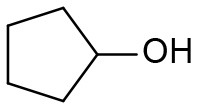	94	90	88
14^e^	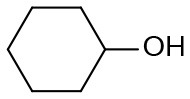	92	88	85
15^e^	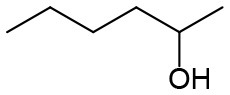	91	90	80
16^e^	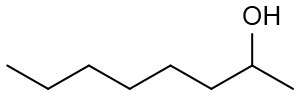	93	91	81

a *Reaction conditions: alcohol (1.0 mmol), catalyst 1c (0.5 mol%), solvent (2.0 mL)*.

b *Conversion was determined by GC*.

c *Isolated yields*.

d *TOF was calculated at 30% conversion of alcohol*.

e *Reaction time 3 h, yield was determined by GC*.

Moreover, the oxidation of primary alcohols was also tested in the standard conditions and the results were summarized in Table 4. The reaction of benzyl alcohols bearing electron-rich or electron-deficient substituents in the aromatic ring proceeded efficiently to furnish the corresponding benzaldehyde derivatives in excellent yields (Table 4, entries 1–7). Only trace amount of benzoic acids were detected, which demonstrated the superiority of the present catalytic system in terms of chemoselectivity. 2-Naphthalenemethanol showed satisfactory reactivity to provide 2-naphthaldehyde in >90% yield (Table 4, entry 8). The substrate having sensitive group (internal alkene) was also tolerated in this system, the carbon-carbon double was preserved in the final product (Table 4, entry 9). While the catalytic system displayed a diminished activity for oxidation of heterocyclic primary alcohols such as 2-furanmethanol and 2-thiophenemethanol, and <70% yields of the target products were obtained under optimized condition. This outcome can be explained by the strong coordination ability of heteroatoms with Ru centers, which led to the deactivation of the catalyst. Delightfully, decent yields of the heterocyclic products could be obtained by simply increasing the catalyst loading of **1c** to 1.5 mol% (Table 4, entries 10–12).

**Table 4:** Oxidation of various primary alcohols to aldehydes catalyzed by 1c^a^.

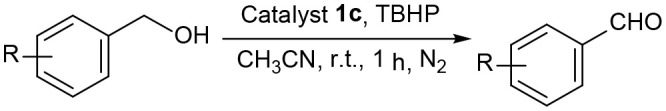				
**Entry**	**Alcohol**	**Conv.^b^ (%)**	**Yield^c^ (%)**	**TOF^d^ (h^−1^)**
1	R = H	94	88	279
2	R = 4-Me	95	90	286
3	R = 3-Me	97	90	290
4	R = 4-OMe	99	93	302
5	R = 4-Cl	94	85	271
6	R = 4-Br	95	90	276
7	R = 4-NO_2_	97	91	277
8	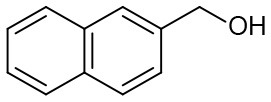	98	92	300
9	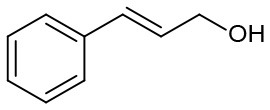	95	90	287
10^e^	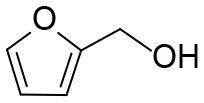	87	82	88
11^e^	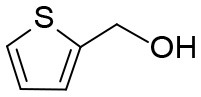	89	85	92
12^e^	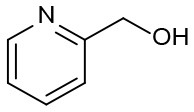	84	81	83

a *Reaction conditions: alcohol (1.0 mmol), catalyst 1c (0.5 mol%), solvent (2.0 mL)*.

b *Conversion was determined by GC*.

c *Isolated yields*.

d *TOF was calculated at 30% conversion of alcohol*.

e *1.5 mol% of 1c was used*.

**Scheme 2:**
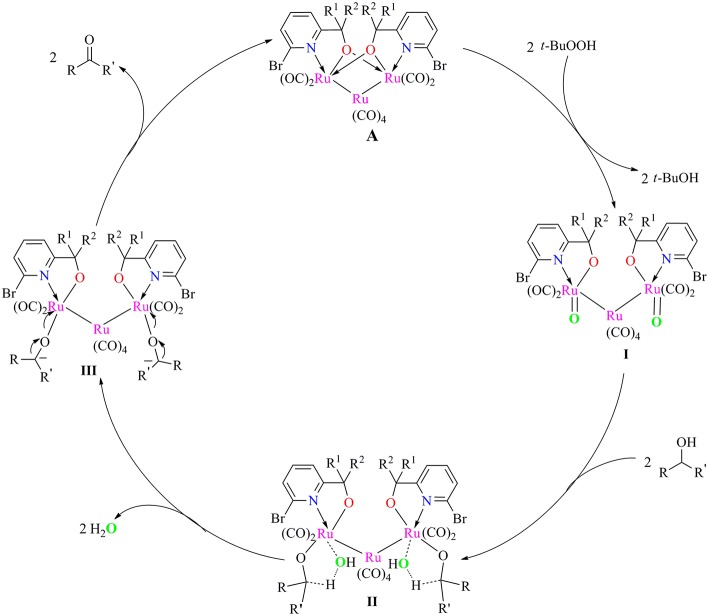
Proposed mechanism for alcohol oxidation catalyzed by Ru/TBHP system.

Based on our preliminary data and related Ru-catalyzed alcohol oxidation processes, a plausible inner-sphere mechanism for alcohol oxidation catalyzed by present ruthenium carbonyl complexes/TBHP system is proposed in Scheme 2. First, the catalyst **A** reacted with two molecules of TBHP to give Ru-oxide species **I** and ^*t*^BuOH. Subsequent reaction of intermediate **I** with alcohol to form a five-membered ring transient state **II**, which then released water to give the alkoxide species **III**. Finally, the break of Ru-O bond in this intermediate occurred to regenerate catalyst **A** for next catalytic cycle and afforded the target carbonyl product.

## Conclusions

In summary, a series of ruthenium carbonyl complexes bearing pyridine-alkoxide ligands were synthesized and exhibited excellent catalytic activity for the oxidation of both primary and secondary alcohol substrates, showing broad substrate scope. Of particular note was the effective oxidation of primary alcohols to desired aldehydes without over-oxidation, displaying good chemoselectivity. A striking advantage associated with this catalytic system is that the oxidation reaction can be completed within only 1 h at room temperature for most cases, which is far more efficient than previously reported Ru/TBHP or Ru/NMO systems.

## Data Availability

The raw data supporting the conclusions of this manuscript will be made available by the authors, without undue reservation, to any qualified researcher.

## Author Contributions

XYa and XYu were response for the synthesis and characterization of Ru complexes and catalytic experiment. KL conducted the synthesis of pyridine alcohol ligands. ZHan solved the single crystals and drew figures for the complexes. ZHao and JL supervised the whole work, drafted and revised the manuscript.

### Conflict of Interest Statement

The authors declare that the research was conducted in the absence of any commercial or financial relationships that could be construed as a potential conflict of interest.
