# Supplementary material for: Synthesis and Structures of Ruthenium Carbonyl Complexes Bearing Pyridine-Alkoxide Ligands and Their Catalytic Activity in Alcohol Oxidation
Source: Front Chem. 2019 Jun 4;7:394. doi: 10.3389/fchem.2019.00394 (PMC6558070; doi:10.3389/fchem.2019.00394)
Supplement: Supplementary file 1 [file Table_1.DOC]

Supporting Information

**Synthesis and structures of ruthenium carbonyl complexes bearing pyridine-alkoxide ligands and their catalytic activity in alcohol oxidation**

Xinlong Yan‡, Xiaohui Yue‡, Kang Liu, Zhiqiang Hao*, Zhangang Han and Jin Lin*

^†^Hebei Key Laboratory of Organic Functional Molecules, The College of Chemistry and Material Science, Hebei Normal University, Shijiazhuang, China

‡These authors contributed equally to this work.

*** Correspondence:**Corresponding Author
[linjin64@126.com](mailto:linjin64@126.com), haozhiqiang1001@163.com.

**CONTENT：**

1. Oxidant screening experiments ...............................................................................S2

2. The molecular structures of complexes **1b**, **1d-1f** and **1h**........................................S3-S5

3. Crystal data and structure refinement for **1a-1h**.......................................................S6-S9

4. NMR spectra of the complexes **1a-1h**......................................................................S10-S17

5. NMR date of all the products...................................................................................S18-S23

**1. Oxidant screening experiments**

**Table S1** 1-phenylethanol oxidation catalyzed by complex **1a** with various of oxidants.^a^

| Entry | Catalyst  (%) | Oxidant | Yield^b^  (%) |
| --- | --- | --- | --- |
| 1 | 2.0 | NMO | 67 |
| 2 | 2.0 | H_2_O_2_ | 38 |
| 3 | 2.0 | Bu*^t^*OOH | 75 |
| 4 | 2.0 | TEMPO | 52 |
| ^a^ Reaction conditions: 1-phenylethanol (1.0 mmol), oxidant (3.0 mmol), toluene (3.0 mL), reaction time 3 h. ^b^ Yield was determined by GC. | | | |

**2. The molecular structures of complexes 1b, 1d-1f and 1h**

**
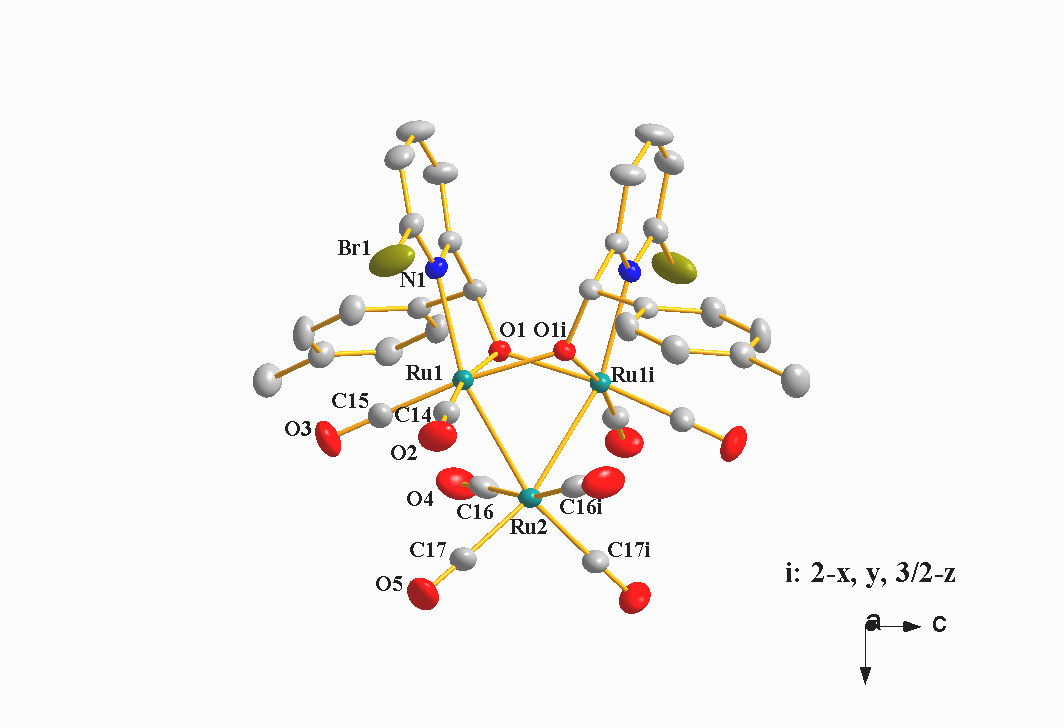
**

**Figure S1.** Perspective view of **1b** with thermal ellipsoids are drawn at the 30% probability level. Hydrogens have been omitted for clarity. The selected bond lengths (Å) and angles (°): Ru(1)-O(1) 2.080(2), Ru(1)-O(1i) 2.124(2), Ru(1)-N(1) 2.269(3), Ru(1)-Ru(2) 2.7579(4), Ru(2)-Ru(1) 2.7579(4); N(1)-Ru(1)-Ru(2) 156.05(8), Ru(1)-O(1)-Ru(1i) 93.08(8), Ru(1)-O(1)-Ru(1i) 93.08(8), Ru(1)-Ru(2)-Ru(1i) 67.181(16).


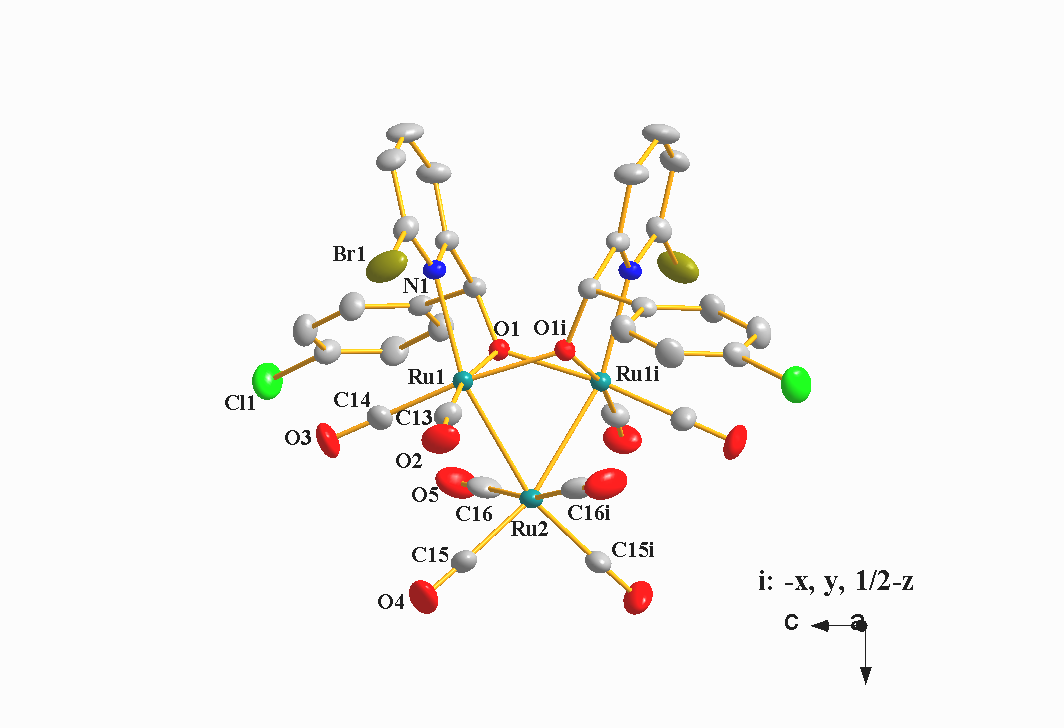


**Figure S2.** Perspective view of **1d** with thermal ellipsoids are drawn at the 30% probability level. Hydrogens have been omitted for clarity. The selected bond lengths (Å) and angles (°): Ru(1)-O(1) 2.079(5), Ru(1)-O(1i) 2.127(5), Ru(1)-Ru(2) 2.7536(10), Ru(2)-Ru(1i) 2.7536(10), Ru(1)-N(1) 2.246(6); Ru(1)-O(1)-Ru(1i) 92.8(2), N(1)-Ru(1)-Ru(2) 156.52(17), Ru(1i)-Ru(2)-Ru(1) 67.17(3).


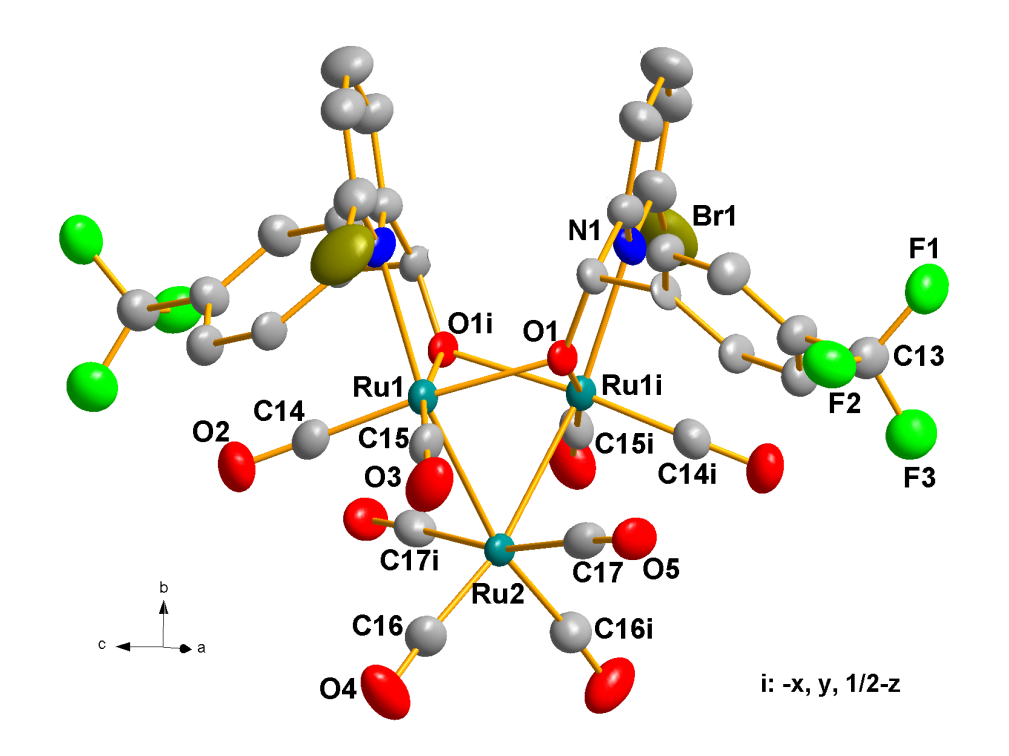


**Figure S3.** Perspective view of **1e** with thermal ellipsoids are drawn at the 30% probability level. Hydrogens have been omitted for clarity. The selected bond lengths (Å) and angles (°): Ru(1)-O(1i) 2.099(10), Ru(1)-O(1) 2.161(10), Ru(1)-N(1i) 2.330(14), Ru(1)-Ru(2) 2.771(2), Ru(2)-Ru(1i) 2.771(2); Ru(1i)-O(1)-Ru(1) 91.6(4), Ru(1)-Ru(2)-Ru(1i) 66.91(7), N(1i)-Ru(1)-Ru(1i) 102.0(4).


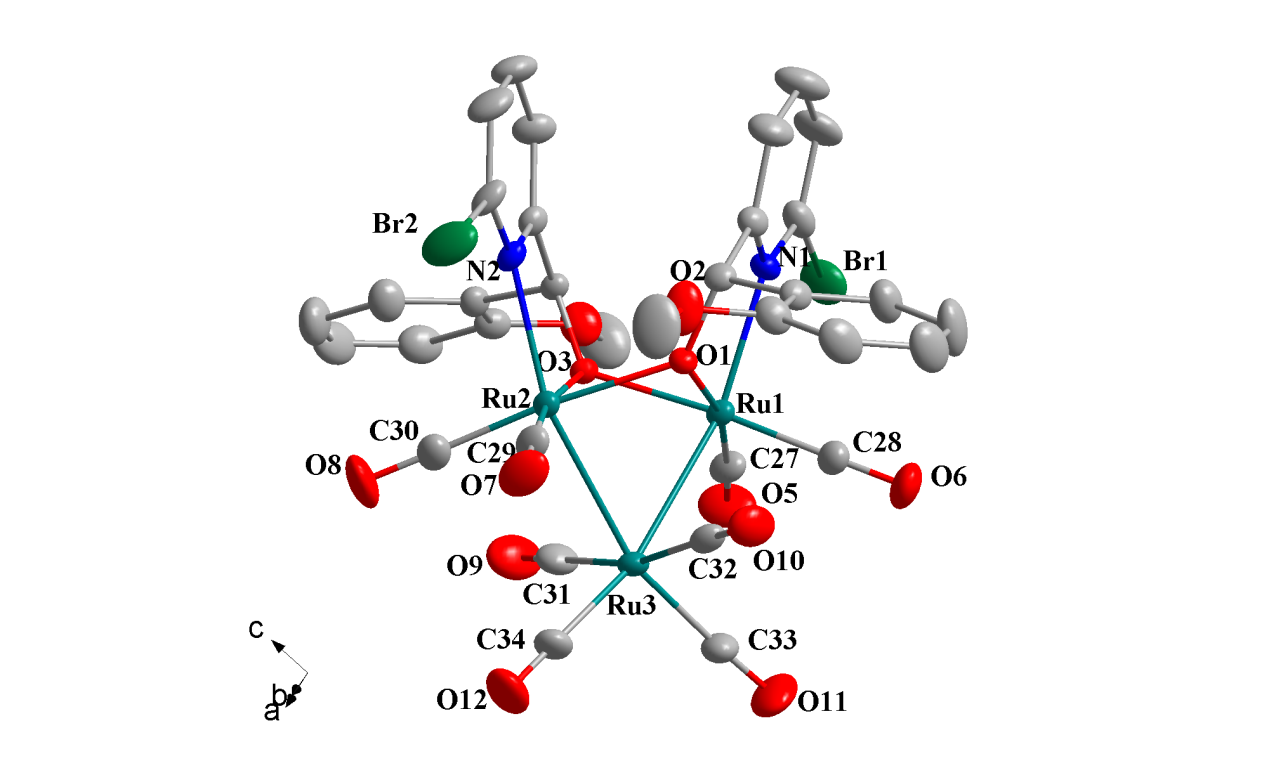


**Figure S4.** Perspective view of **1f** with thermal ellipsoids are drawn at the 30% probability level. Hydrogens have been omitted for clarity. The selected bond lengths (Å) and angles (°): Ru(1)-O(1) 2.078(2), Ru(2)-O(1) 2.128(3), Ru(1)-N(1) 2.258(3), Ru(2)-N(2) 2.247(3), Ru(1)-Ru(3) 2.7578(5), Ru(2)-Ru(3) 2.7544(5); Ru(2)-O(3)-Ru(1) 92.28(10), N(1)-Ru(1)-Ru(3) 157.54(9), Ru(2)-O(3)-Ru(1) 92.28(10), N(2)-Ru(2)-Ru(3) 157.38(9), Ru(2)-Ru(3)-Ru(1) 66.940(13).

**
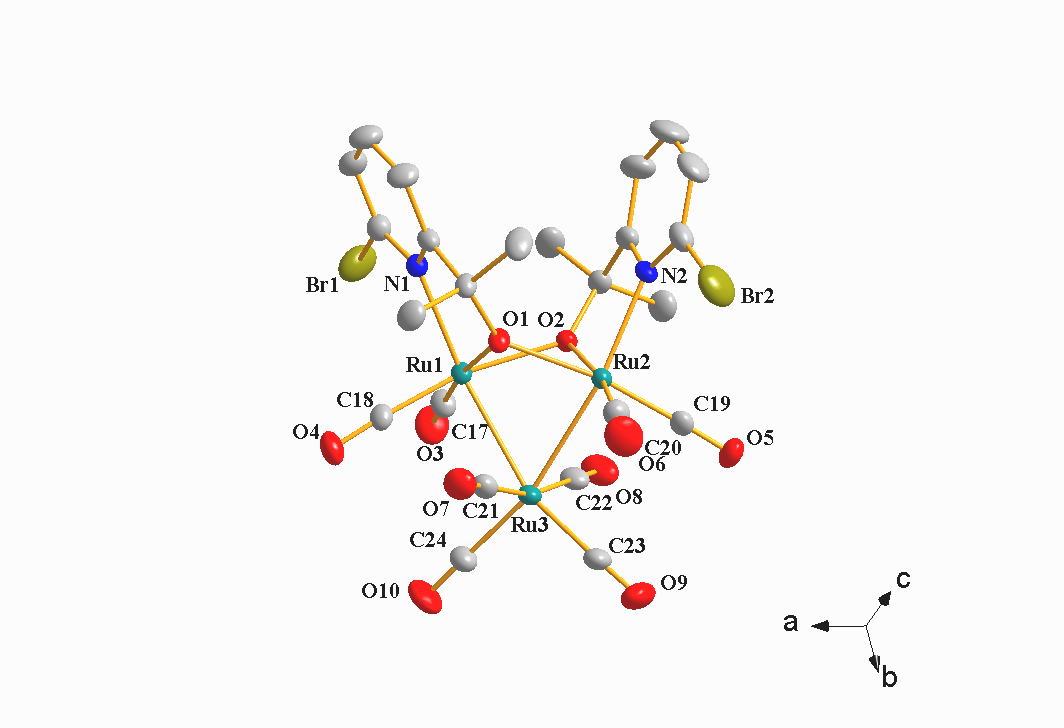
**

**Figure S5.** Perspective view of **1h** with thermal ellipsoids are drawn at the 30% probability level. Hydrogens have been omitted for clarity. The selected bond lengths (Å) and angles (°): Ru(1)-Ru(3) 2.7690(5), Ru(1)-Ru(2) 3.0463(5), Ru(1)-O(1) 2.072(3), Ru(1)-O(2) 2.171(3), Ru(1)-N(1) 2.269(4), Ru(2)-O(2) 2.073(3), Ru(2)-O(1) 2.165(3), Ru(2)-N(2) 2.241(3), Ru(2)-Ru(3) 2.7643(5); N(2)-Ru(2)-Ru(3) 159.39(11), Ru(2)-O(2)-Ru(1) 91.70(11), Ru(3)-Ru(2)-Ru(1) 56.669(13), O(1)-Ru(2)-Ru(3) 82.67(7).

**3. Crystal data and structure refinement for 1a-1h**

**Table S2 Crystal data and structure refinements of complexes 1a-1d.**

| **Complex** | **1a** | **1b** | **1c** | **1d** |
| --- | --- | --- | --- | --- |
| formula | C_32_H_18_Br_2_N_2_O_10_Ru_3_ | C_34_H_22_Br_2_N_2_O_10_Ru_3_ | C_35_H_24_Br_2_Cl_2_N_2_O_12_Ru_3_ | C_32_H_16_Br_2_Cl_2_N_2_O_10_Ru_3_ |
| *Fw* | 1053.51 | 1081.57 | 1198.49 | 1122.4 |
| *T*, K | 298(2) | 298(2) | 298(2) | 298(2) |
| Wavelength(Å) | 0.71073 | 0.71073 | 0.71073 | 0.71073 |
| Crystal system, | Monoclinic | Monoclinic | Monoclinic | Monoclinic |
| space group | *P*2(1)/*n* | *C*2*/c* | *P*2(1)*/n* | *C*2*/c* |
| *a*(Å) | 21.8619(18) | 11.6831(12) | 11.8159(9) | 11.6423(11) |
| *b*(Å) | 16.0265(14) | 16.5574(16) | 22.1210(19) | 16.4542(15) |
| *c*(Å) | 21.8619(18) | 19.8654(18) | 15.9428(12) | 19.7252(17) |
| *a*(**°**) | 90 | 90 | 90 | 90 |
| *β*(**°**) | 113.184(3) | 103.278(2) | 92.002(2) | 103.758(2) |
| *γ*(**°**) | 90 | 90 | 90 | 90 |
| Volume(Å^3^) | 7041.2(10)) | 3740.1(6) | 4164.6(6) | 3670.2(6) |
| *Z* | 8 | 4 | 4 | 4 |
| *D*_calc_(mg/m^3^) | 1.988 | 1.921 | 1.911 | 2.301 |
| *μ*(mm^-1^) | 3.598 | 3.389 | 3.181 | 3.598 |
| *F*(000) | 4048 | 2088 | 2320 | 2152 |
| Crystal size(mm) | 0.42×0.40×0.13 | 0.30×0.25×0.12 | 0.43×0.35×0.30 | 0.32×0.18×0.13 |
| *θ* range(**°**) | 2.27-25.02 | 2.46-25.02 | 2.24-25.02 | 2.48-25.02 |
| Reflections collected | 34587/12382 | 9231/3287 | 20667/7318 | 8853/3207 |
| *R*(int) | 0.0747 | 0.0319 | 0.0296 | 0.0429 |
| Max. and min. transmission | 0.6520/0.3134 | 0.6865/0.4296 | 0.4487/0.3416 | 0.6520/0.3922 |
| Data/restraints/ parameters | 12382/0/883 | 3287/0/232 | 7318/0/511 | 3207/0/231 |
| Goodness of  fit on *F*^2^ | 1.07 | 1.025 | 1.043 | 1.096 |
| *R*_1_, w*R*_2_[*I*>2σ(*I*)] | 0.0605, 0.1238 | 0.0266, 0.0599 | 0.0368, 0.0814 | 0.0525, 0.1486 |
| *R*_1_, w*R*_2_ (all data) | 0.1226, 0.1432 | 0.0402, 0.0652 | 0.0574, 0.0887 | 0.0694, 0.1579 |
| Max. peak/(e.Å^-3^) | 1.526 | 0.747 | 0.558 | 1.771 |
| Mini. peak/(e.Å^-3^) | -1.382 | -0.6 | -0.922 | -1.418 |
| CCDC | 1554244 | 1561567 | 1554281 | 1561568 |

**Table S3 Crystal data and structure refinements of complexes 1e-1h.**

| **Complex** | **1e** | **1f** | **1g** | **1h** |
| --- | --- | --- | --- | --- |
| formula | C_34_H_16_Br_2_F_6_N_2_O_10_Ru_3_ | C_34_H_22_Br_2_N_2_O_12_Ru_3_ | C_34_H_16_Br_2_F_6_N_2_O_10_Ru_3_ | C_24.50_H_19_Br_2_ClN_2_O_10_Ru_3_ |
| *Fw* | 1189.52 | 1113.57 | 1189.52 | 999.9 |
| *T*, K | 298(2) | 298(2) | 273(2) | 298(2) |
| Wavelength(Å) | 0.71073 | 0.71073 | 0.71073 | 0.71073 |
| Crystal system, | Monoclinic | Monoclinic | Tetragonal | Monoclinic |
| space group | *C*2/*c* | *P*2(1)/*n* | *P*4(3)2(1)2 | *P*2(1)*/n* |
| *a*(Å) | 22.947(2) | 10.5890(9) | 10.7588(3) | 8.9866(8) |
| *b*(Å) | 12.7831(12) | 16.6908(13) | 10.7588(3) | 18.9766(15) |
| *c*(Å) | 16.0819(16) | 22.2028(18) | 33.205(2) | 19.3186(17) |
| *a*(**°**) | 90 | 90 | 90 | 90 |
| *β*(**°**) | 122.360(3) | 100.665(2) | 90 | 99.894(2) |
| *γ*(**°**) | 90 | 90 | 90 | 90 |
| Volume(Å^3^) | 3984.8(7) | 3856.3(5) | 3843.6(3) | 3245.5(5) |
| *Z* | 4 | 4 | 4 | 4 |
| *D*_calc_(mg/m^3^) | 1.983 | 1.918 | 2.056 | 2.046 |
| *μ*(mm^-1^) | 3.212 | 3.294 | 3.33 | 3.975 |
| *F*(000) | 2280 | 2152 | 2280 | 1916 |
| Crystal size(mm) | 0.15×0.10×0.06 | 0.35×0.33×0.32 | 9.07×6.70×4.50 | 0.27×0.20×0.16 |
| *θ* range(**°**) | 2.75-25.02 | 2.31-25.02 | 2.26-28.28 | 2.39-25.02 |
| Reflections collected | 9187/3448 | 19033/6791 | 54420/4785 | 16071/5693 |
| *R*(int) | 0.1666 | 0.0398 | 0.0492 | 0.028 |
| Completeness to *θ* | 97.90% | 99.80% | 99.90% | 99.40% |
| Max. and min. transmission | 0.8307/0.6444 | 0.4187/0.3919 | 0.0235/0.0137 | 0.5688/0.4133 |
| Data/restraints/ parameters | 3448/0/286 | 6791/0/480 | 4785/0/253 | 5693/0/401 |
| Goodness of  fit on *F*^2^ | 1.083 | 1.009 | 0.894 | 1.024 |
| *R*_1_, w*R*_2_[*I*>2σ(*I*)] | 0.0970, 0.1995 | 0.0340, 0.0676 | 0.0500, 0.1398 | 0.0318, 0.0684 |
| *R*_1_, w*R*_2_ (all data) | 0.1954, 0.2330 | 0.0584, 0.0741 | 0.0531, 0.1448 | 0.0500, 0.0736 |
| Max. peak/(e.Å^-3^) | 1.85 | 0.553 | 0.811 | 0.742 |
| Mini. peak/(e.Å^-3^) | -1.112 | -0.778 | -1.765 | -0.897 |
| CCDC | 1559099 | 1849161 | 1883760 | 1561569 |

**4. NMR spectra of the complexes 1a-1h**

1. (6-bromopyCHC_6_H_5_O)_2_Ru_3_(CO)_8_ (**1a**)


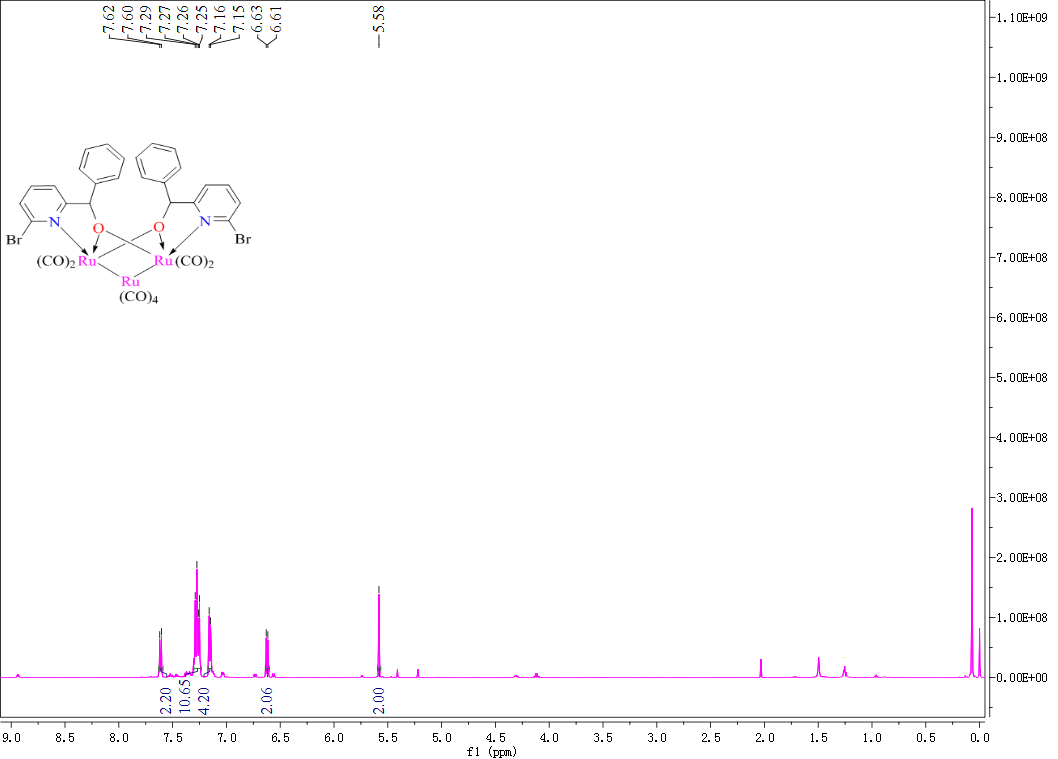


^1^H NMR


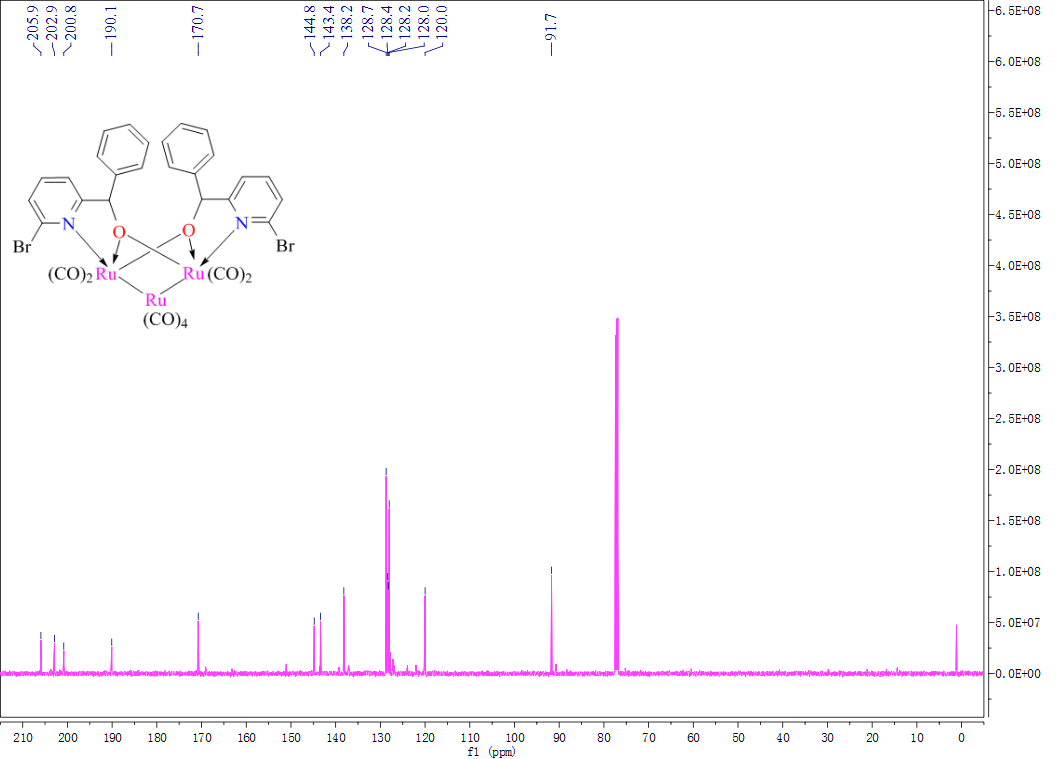


^13^C NMR

2. [6-bromopyCH(4-MeC_6_H_4_)O]_2_Ru_3_(CO)_8_ (**1b**)


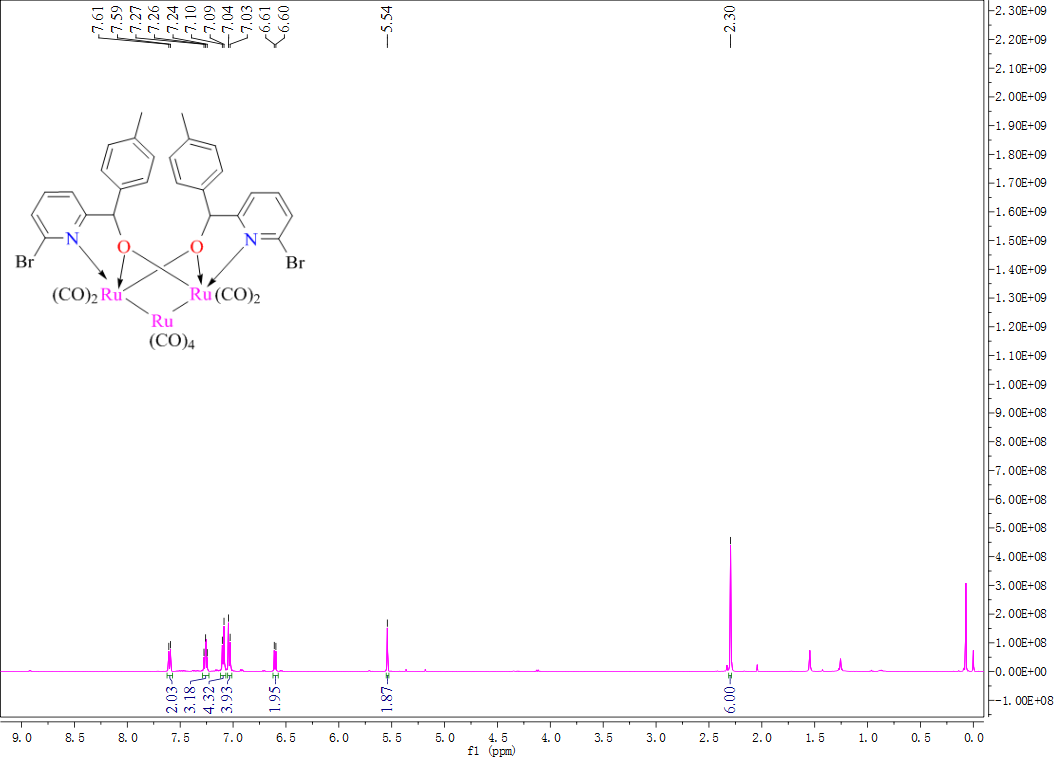


^1^H NMR


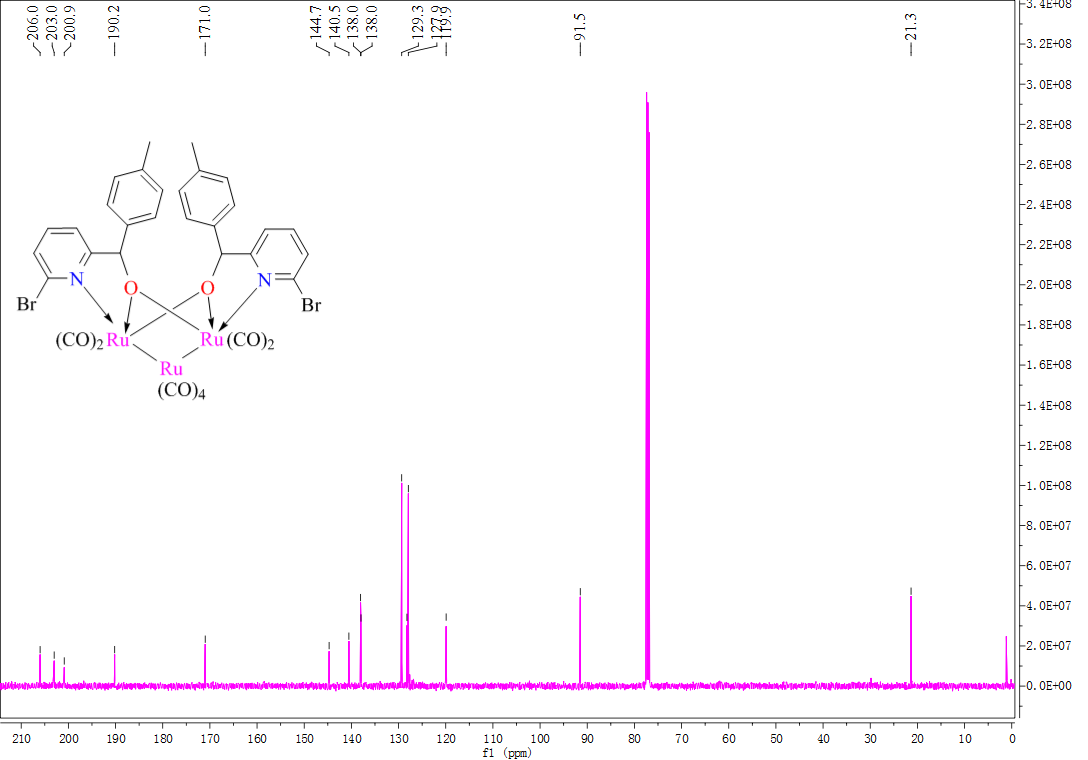


^13^C NMR

3. [6-bromopyCH(4-OMeC_6_H_4_)O]_2_Ru_3_(CO)_8_ (**1c**)


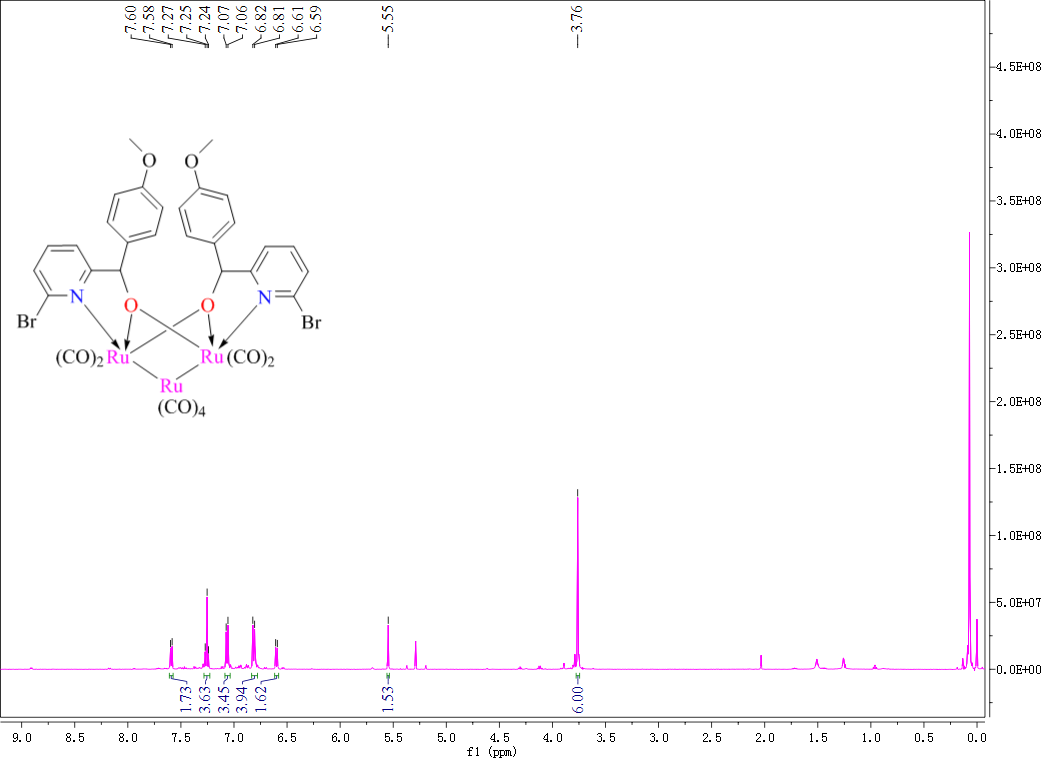


^1^H NMR


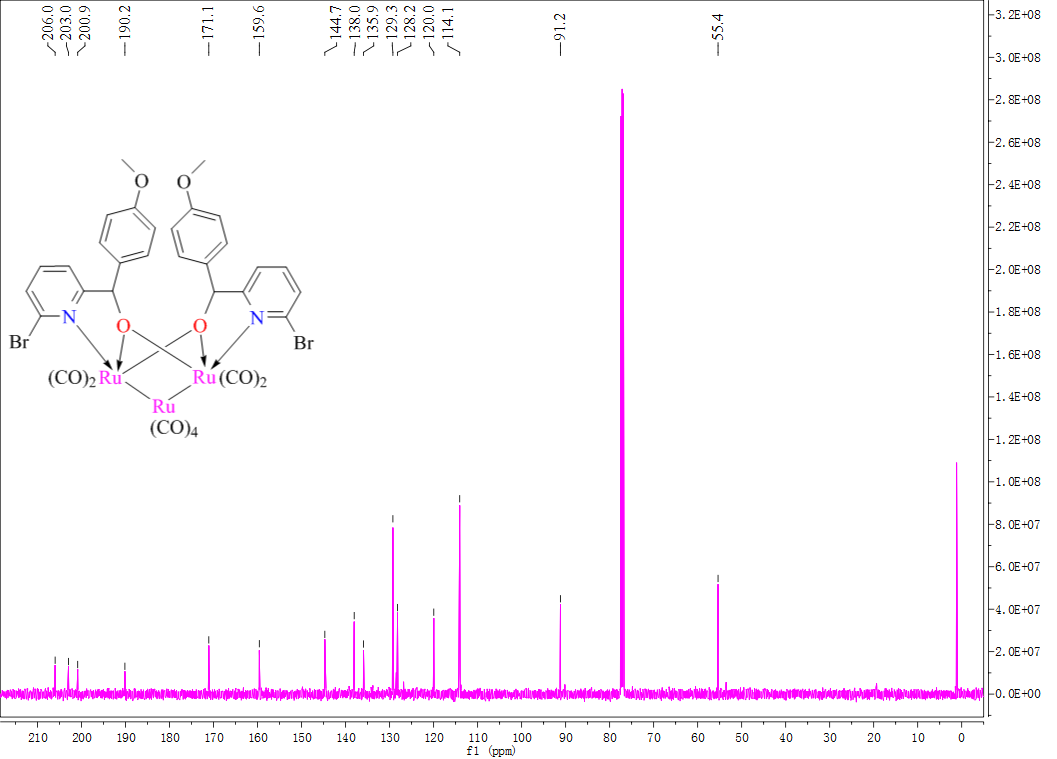


^13^C NMR

4. [6-bromopyCH(4-ClC_6_H_4_)O]_2_Ru_3_(CO)_8_ (**1d**)


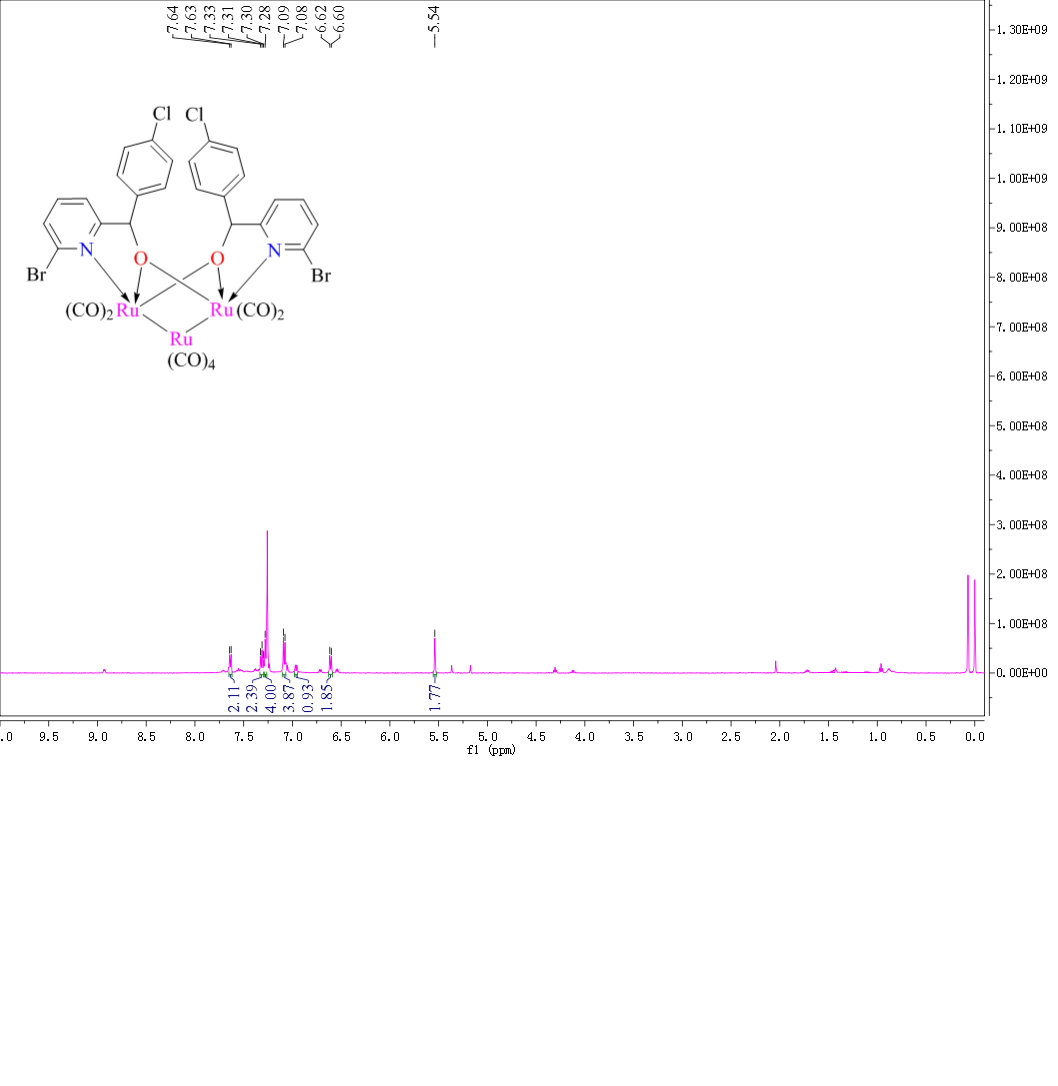


^1^H NMR


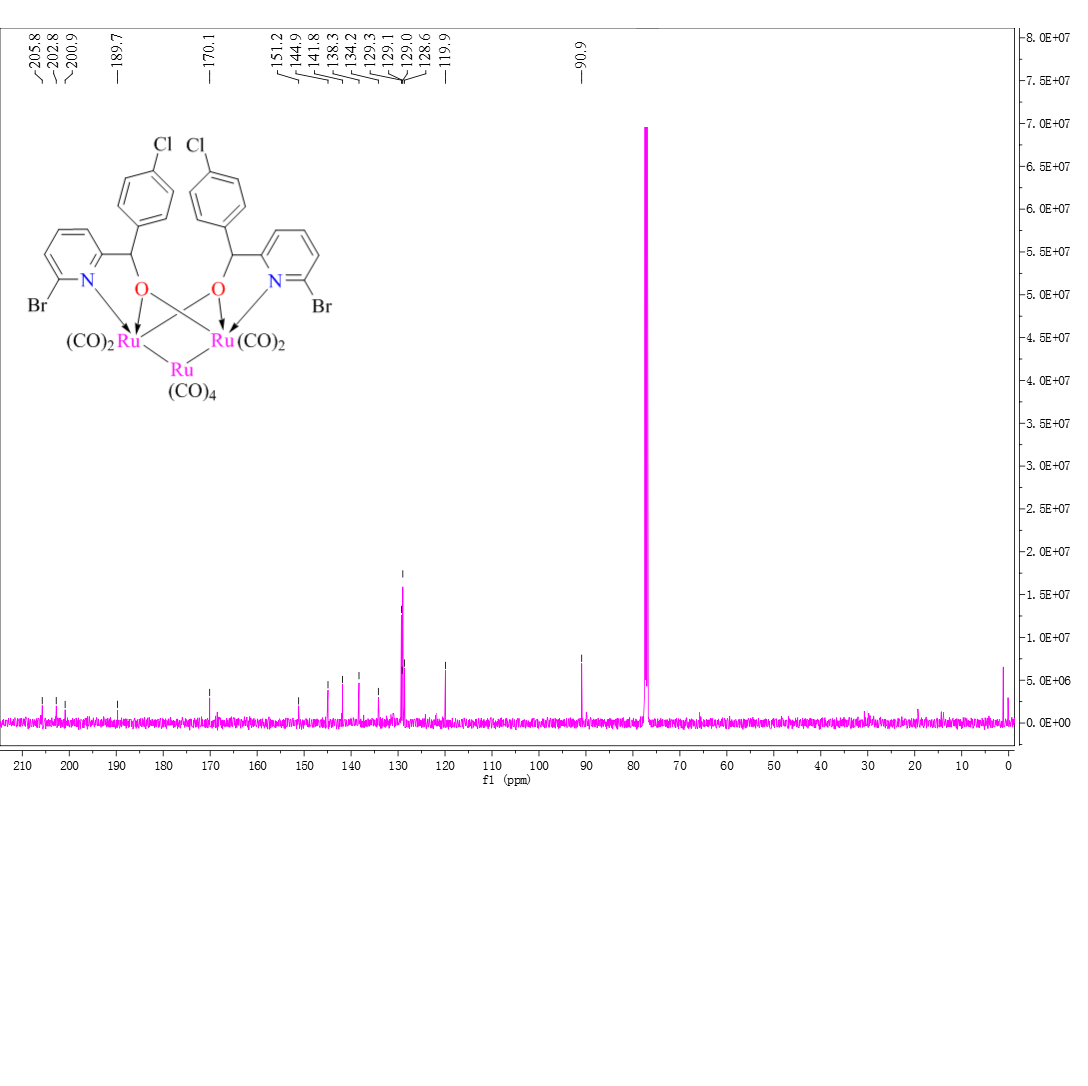


^13^C NMR

5. [6-bromopyCH(4-CF_3_C_6_H_4_)O]_2_Ru_3_(CO)_8_ (**1e**)


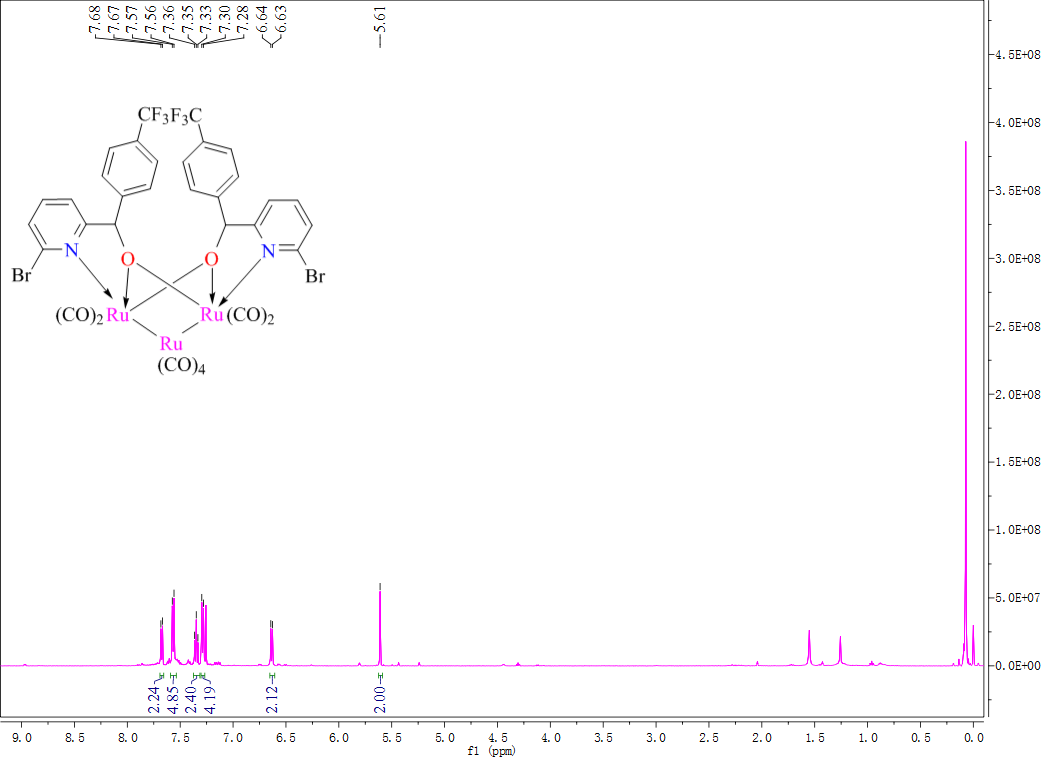


^1^H NMR


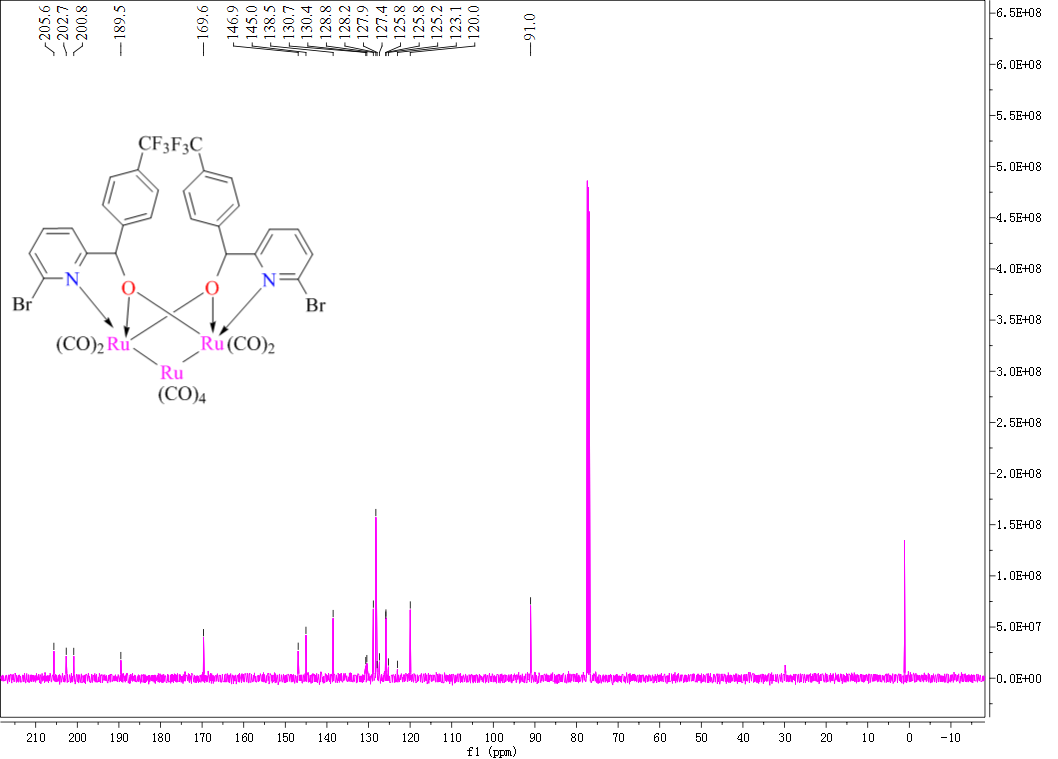


^13^C NMR

6. [6-bromopyCH(2-OCH_3_C_6_H_4_)O]_2_Ru_3_(CO)_8_ (**1f**)


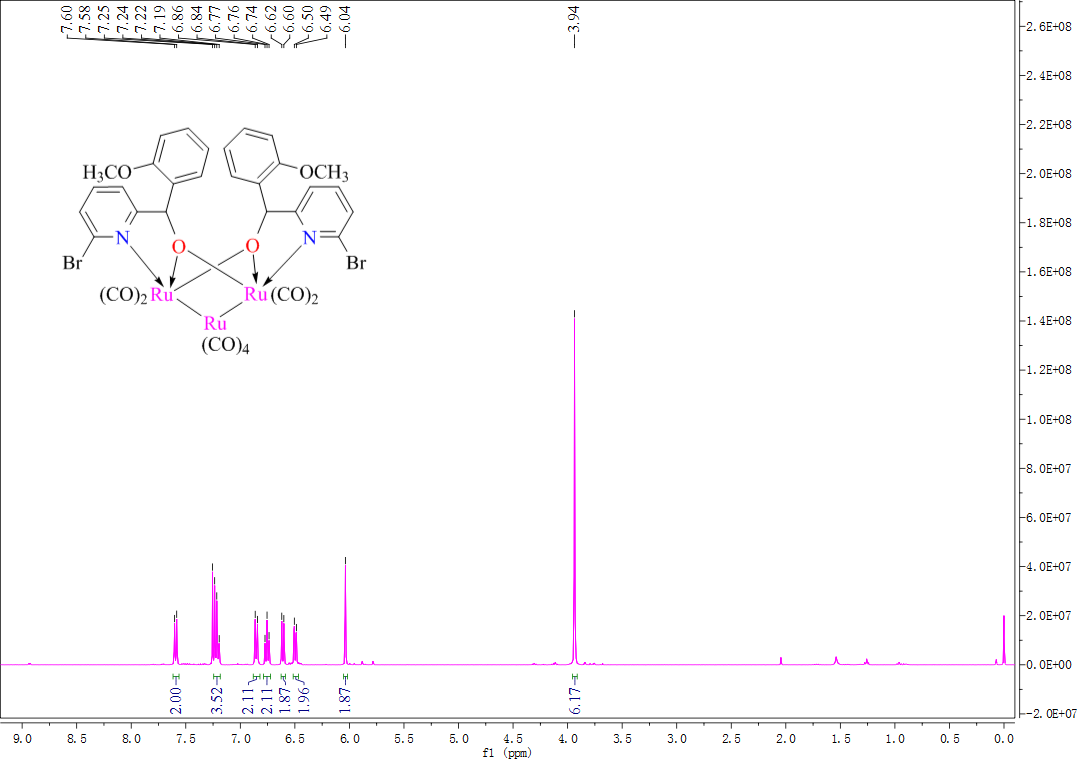


^1^H NMR


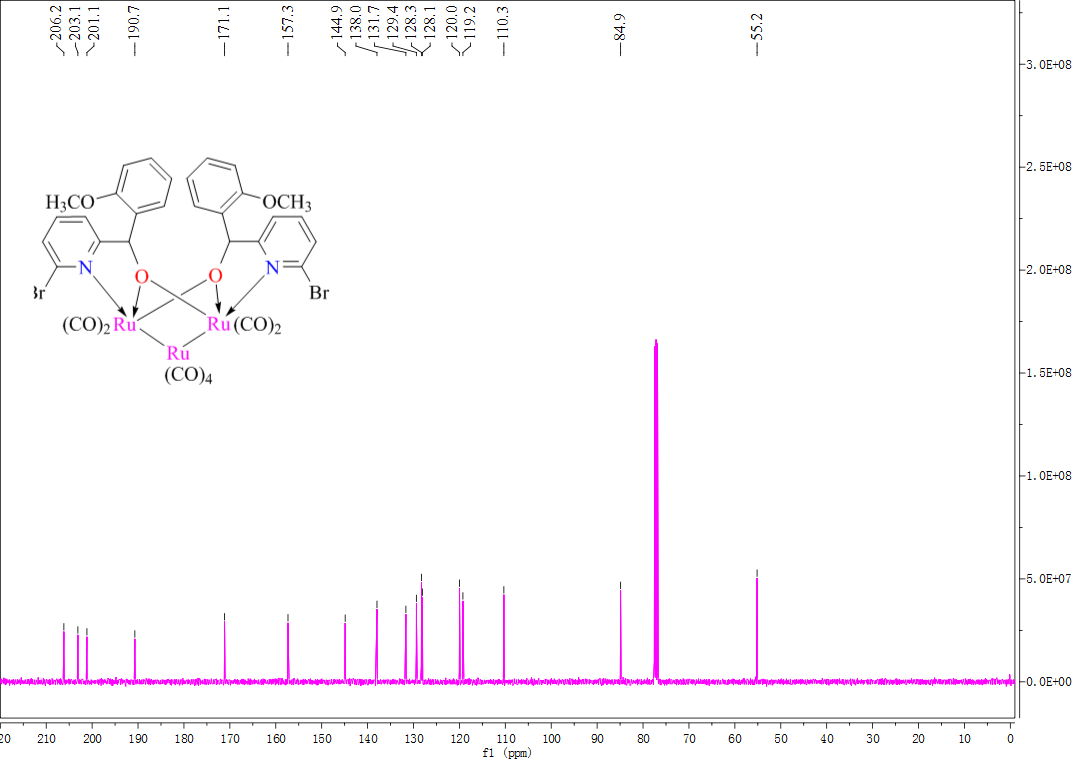


^13^C NMR

7. [6-bromopyCH(2-CF_3_C_6_H_4_)O]_2_Ru_3_(CO)_8_ (**1g**)


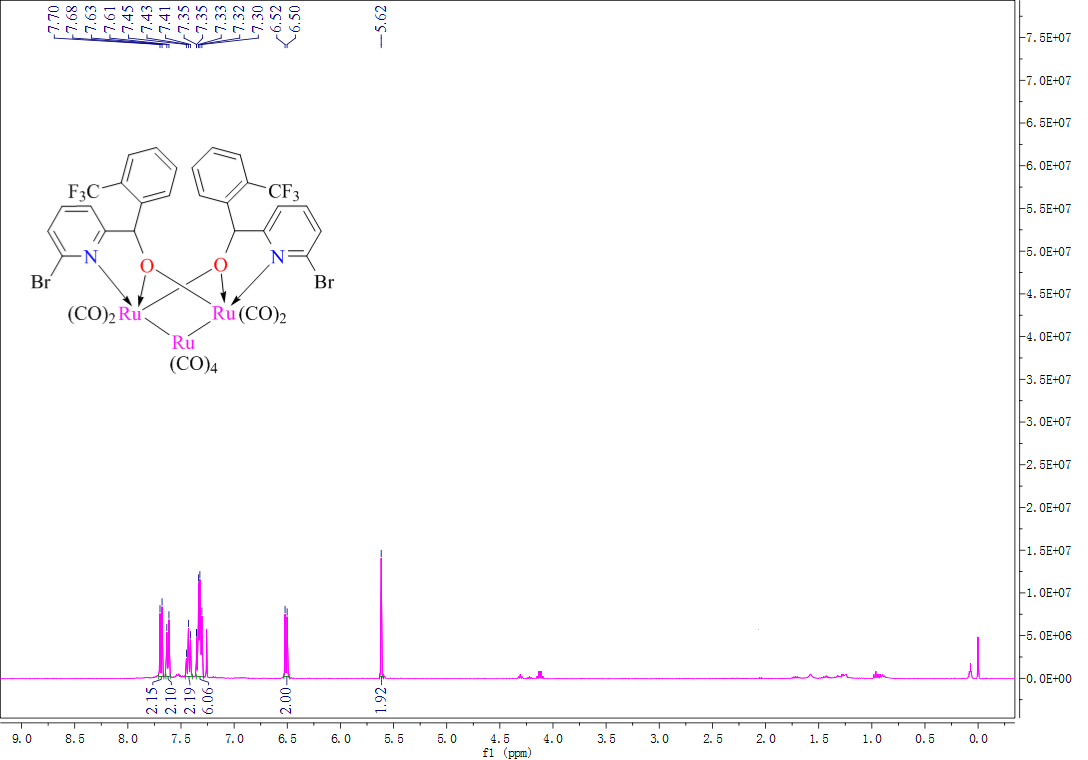


^1^H NMR


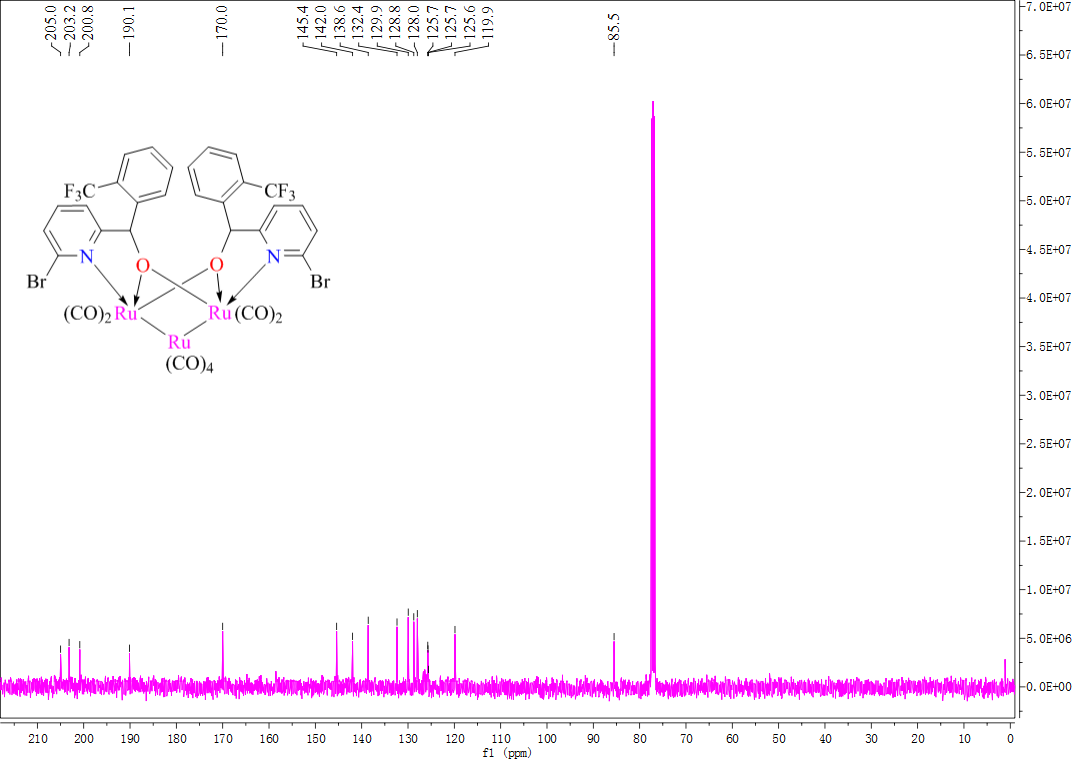


^13^C NMR

8. [6-bromopyCH(CH_3_)_2_O]_2_Ru_3_(CO)_8_ (**1h**)


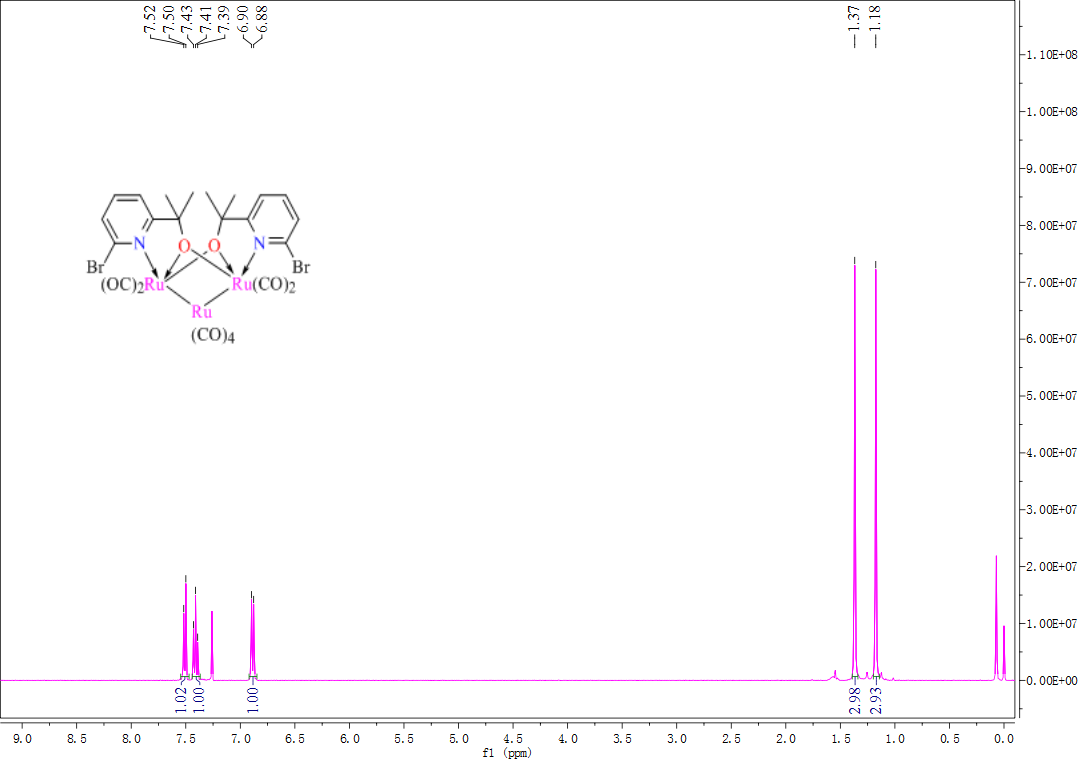


^1^H NMR


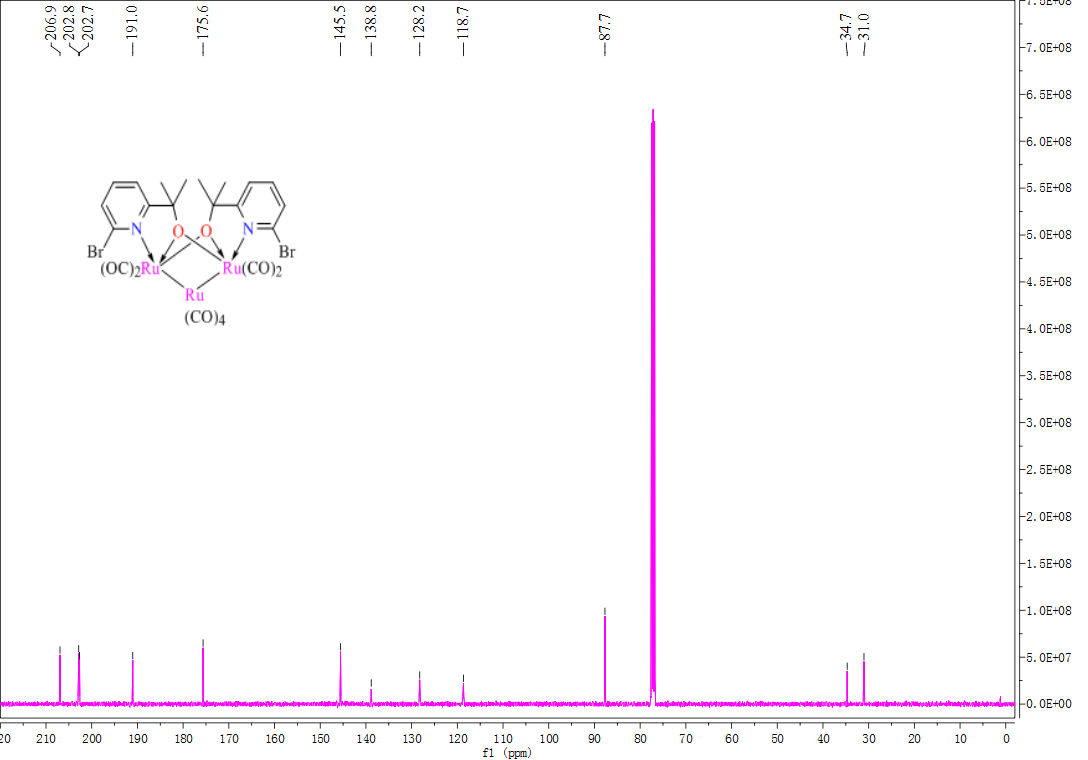


^13^C NMR

**5. NMR date of all the products**

1. benzaldehyde

^1^H NMR (CDCl_3_, 500 MHz, 298 K): δ 10.02 (s, 1 H, CHO), 7.88 (d, *J* = 7 Hz, 2 H, Ph-H), 7.62 (t, *J* = 10 Hz, 1 H, Ph-H), 7.53 (t, *J* = 7.6 Hz, 2 H, Ph-H) ppm. ^13^C NMR (CDCl_3_, 125 MHz, 298 K): δ 192.5, 136.6, 134.6, 129.8, 129.1 ppm.

2. 4-methoxybenzaldehyde

^1^H NMR (CDCl_3_, 500 MHz, 298 K): δ 9.88 (s, 1 H, CHO), 7.84 (d, *J* = 8.6 Hz, 2 H, C_6_H_4_), 7.00 (d, *J* = 8.6 Hz, 2 H, C_6_H_4_), 3.88 (s, 3 H, OCH_3_) ppm. ^13^C NMR (CDCl_3_, 125 MHz, 298 K): δ 188.9, 162.8, 130.4, 130.1, 112.5, 53.7 ppm.

3. 4-methylbenzaldehyde

^1^H NMR (CDCl_3_, 500 MHz, 298 K): δ 9.93 (s, 1 H, CHO), 7.74 (d, *J* = 8.0 Hz, 2 H, C_6_H_4_), 7.29 (d, *J* = 8.0 Hz, 2 H, C_6_H_4_), 2.40 (s, 3 H, CH_3_) ppm. ^13^C NMR (CDCl_3_, 125 MHz, 298 K): δ 192.0, 145.6, 134.2, 129.9, 129.7, 21.9 ppm.

4. 3-methylbenzaldehyde

^1^H NMR (CDCl_3_, 500 MHz, 298 K): δ 9.96 (s, 1 H, CHO), 7.64-7.68 (m, 2 H, C_6_H_4_), 7.37-7.41 (m, 2 H, C_6_H_4_), 2.41 (s, 3 H, CH_3_) ppm. ^13^C NMR (CDCl_3_, 125 MHz, 298 K): δ 192.6, 138.9, 136.5, 135.3, 130.0, 128.9, 127.2, 21.2 ppm.

5. 4-chlorobenzaldehyde

^1^H NMR (CDCl_3_, 500 MHz, 298 K): δ 9.98 (s, 1 H, CHO), 7.82 (d, *J* = 8.5 Hz, 2 H, C_6_H_4_), 7.51 (d, *J* = 8.4 Hz, 2 H, C_6_H_4_) ppm. ^13^C NMR (CDCl_3_, 125 MHz, 298 K): δ 190.9, 141.1, 134.9, 131.0, 129.6 ppm.

6. 4-nitrobenzaldehyde

^1^H NMR (CDCl_3_, 500 MHz, 298 K): δ 10.15 (s, 1 H, CHO), 8.39 (d, *J* = 8.7 Hz, 2 H, C_6_H_4_), 8.07 (d, *J* = 8.8Hz, 2 H, C_6_H_4_) ppm. ^13^C NMR (CDCl_3_, 125 MHz, 298 K): δ 190.5, 151.2, 140.2, 130.6, 124.4 ppm.

7. 1-naphthaldehyde

^1^H NMR (CDCl_3_, 500 MHz, 298 K): δ 10.41 (s, 1 H, CHO), 9.28 (d, *J* = 8.6 Hz, 1 H, C_8_H_7_), 8.09 (d, *J* = 8.2 Hz, 1 H, C_8_H_7_), 7.99 (d, *J* = 7.0, 1 H, C_8_H_7_), 7.91 (d, *J* = 9.3 Hz, 1 H, C_8_H_7_), 7.71 (t, *J* = 8.6 Hz, 1 H, C_8_H_7_), 7.59-7.64 (m, 2 H, C_8_H_7_) ppm. ^13^C NMR (CDCl_3_, 125 MHz, 298 K): δ 193.4, 136.5, 135.2, 133.8, 131.5, 130.6, 129.1, 128.5, 127.0, 124.9, 123.7 ppm.

8. furan-2-carbaldehyde

^1^H NMR (CDCl_3_, 500 MHz, 298 K): δ 9.65 (s, 1 H, CHO), 7.68 (s, 1 H, furan-CH), 7.24 (d, *J* = 3.6 Hz, 1 H, furan-CH), 6.59 (d, *J* = 5.1 Hz, 1 H, furan-CH) ppm. ^13^C NMR (CDCl_3_, 125 MHz, 298 K): δ 178.0, 153.1, 148.2, 112.7, 112.6 ppm.

9. thiophene-2-carbaldehyde

^1^H NMR (CDCl_3_, 500 MHz, 298 K): δ 9.95 (s, 1 H, CHO), 7.76-7.79 (m, 2 H, thiophene-CH), 7.22 (t, *J* = 3.6 Hz, 1 H, thiophene-CH) ppm. ^13^C NMR (CDCl_3_, 125 MHz, 298 K): δ 183.0, 136.5, 135.3, 128.5, 128.4 ppm.

10. Cinnamaldehyde

^1^H NMR (CDCl_3_, 500 MHz, 298 K): δ 9.70 (d, *J* = 7.7 Hz, 1 H, CHO), 7.57-7.55 (m, 2 H, C_6_H_5_), 7.45-7.40 (m, 4 H, C_6_H_5_, = CH), 6.75 -6.69 (m, 1 H, =CH). ^13^C NMR (CDCl_3_, 125 MHz, 298 K): δ 193.8, 152.9, 133.9, 131.3, 128.8, 128.5, 125.7.

11. Acetophenone

^1^H NMR (CDCl_3_, 500 MHz, 298 K): *δ* 7.90 (d, *J* = 7.5 Hz, 2 H, Ph-H), 7.50 (t, *J* = 7.5 Hz, 1 H, Ph-H), 7.40 (t, *J* = 7.5 Hz, 2 H, Ph-H), 2.54 (s, 3 H, CH_3_) ppm. ^13^C NMR (CDCl_3_, 125 MHz, 298 K): *δ* 198.0, 137.0, 133.0, 128.6, 128.2, 26.5 ppm.

12. 1-(p-tolyl)ethanone

^1^H NMR (CDCl_3_, 500 MHz, 298 K): δ 7.85 (d, *J* = 8.0 Hz, 2 H, C_6_H_4_), 7.25 (d, *J* = 8.0 Hz, 2 H, C_6_H_4_), 2.57 (s, 3 H, CH_3_), 2.41 (s, 3 H, CH_3_) ppm. ^13^C NMR (CDCl_3_, 125 MHz, 298 K): δ 198.0, 144.0, 134.8, 129.3, 128.5, 26.6, 21.7 ppm.

13. 1-(m-tolyl)ethanone

^1^H NMR (CDCl_3_, 500 MHz, 298 K): δ 7.74-7.77 (m, 2 H, C_6_H_4_), 7.32-7.39 (m, 2 H, C_6_H_4_), 2.59 (s, 3 H, CH_3_), 2.41 (s, 3 H, CH_3_) ppm. ^13^C NMR (CDCl_3_, 125 MHz, 298 K): δ 197.4, 137.3, 136.1, 132.8, 127.8, 127.4, 124.6, 26.5, 20.3 ppm.

14. 1-(4-methoxyphenyl)ethanone

^1^H NMR (CDCl_3_, 500 MHz, 298 K): *δ* 7.91 (d, *J* = 8.0 Hz, 2 H, C_6_H_4_), 6.91 (d, *J* = 8.0 Hz, 2 H, C_6_H_4_), 3.84 (s, 3 H, OCH_3_), 2.53 (s, 3 H, CH_3_) ppm. ^13^C NMR (CDCl_3_, 125 MHz, 298 K): *δ* 196.8, 163.6, 130.6, 130.4, 113.8, 55.5, 26.4 ppm.

15. 1-(4-chlorophenyl)ethanone

^1^H NMR (CDCl_3_, 500 MHz, 298 K): *δ* 7.82 (d, *J* = 8.5 Hz, 2 H, C_6_H_4_), 7.35 (d, *J* = 9.0 Hz, 2 H, C_6_H_4_), 2.52 (s, 3 H, CH_3_) ppm. ^13^C NMR (CDCl_3_, 125 MHz, 298 K): *δ* 196.7, 139.5, 135.4, 129.7, 128.8, 26.5 ppm.

16. 1-(4-bromophenyl)ethanone

^1^H NMR (CDCl_3_, 500 MHz, 298 K): *δ* 7.81 (d, *J* = 8.5 Hz, 2 H, C_6_H_4_), 7.59 (d, *J* = 8.5 Hz, 2 H, C_6_H_4_), 2.58 (s, 3 H, CH_3_) ppm. ^13^C NMR (CDCl_3_, 125 MHz, 298 K): *δ* 197.0, 135.9, 132.0, 123.0, 128.4, 26.6 ppm.

17. 1-(3-bromophenyl)ethanone

^1^H NMR (CDCl_3_, 500 MHz, 298 K): δ 8.06 (s, 1 H, C_6_H_4_), 7.86 (d, J = 7 Hz, 1 H, C_6_H_4_), 7.67 (d, *J* = 8 Hz, 1 H, C_6_H_4_), 7.33 (t, *J* = 7.5 Hz, 1 H, C_6_H_4_), 2.58 (s, 3 H, CH_3_); ^13^C NMR (CDCl_3_, 125 MHz, 298 K): δ 196.5, 138.8, 135.9, 131.3, 130.2, 126.9, 122.9, 26.6.

18. 1-(4-(trifluoromethyl)phenyl)ethanone

^1^H NMR (CDCl_3_, 500 MHz, 298 K): *δ* 8.06 (d, *J* = 8.0 Hz, 2 H, C_6_H_4_), 7.71 (d, *J* = 8.0 Hz, 2 H, C_6_H_4_), 2.64 (s, 3 H, CH_3_) ppm. ^13^C NMR (CDCl_3_, 125 MHz, 298 K): *δ* 196.9, 139.7, 134.3(q, *J*_C-F_ = 32.5 Hz), 128.6, 125.7 (q, *J*_C-F_ = 3.8 Hz), 122.5 (q, *J*_C-F_ = 276.3 Hz), 26.7 ppm.

19. 1-(naphthalen-2-yl)ethanone

^1^H NMR (CDCl_3_, 500 MHz, 298 K): δ 8.45 (s, 1 H, C_8_H_7_), 8.03 (d, *J* = 9 Hz, 1 H, C_8_*H*_7_), 7.95 (s, 1 H, C_8_*H*_7_), 7.85-7.89 (m, 2 H, C_8_H_7_), 7.53-7.61 (m, 2 H, C_8_H_7_), 2.71 (s, 3 H, CH_3_) ppm. ^13^C NMR (CDCl_3_, 125 MHz, 298 K): δ 198.1, 135.6, 134.6, 132.5, 130.2, 129.6, 128.5, 128.4, 127.9, 126.8, 123.9, 26.7 ppm.

20. benzophenone

^1^H NMR (CDCl_3_, 500 MHz, 298 K): *δ* 7.81 (t, *J* = 9.5 Hz, 4 H, Ph-H), 7.59 (t, *J* = 7.5 Hz, 2 H, Ph-H), 7.48 (d, *J* = 7.5 Hz, 4 H, Ph-H) ppm. ^13^C NMR (CDCl_3_, 125 MHz, 298 K): δ 196.7, 137.6, 132.4, 130.1, 128.3 ppm.

21. 9H-fluoren-9-one

^1^H NMR (CDCl_3_, 500 MHz, 298 K): δ 7.69 (d, *J* = 7.4 Hz, 2 H, C_6_H_4_), 7.55-7.49 (m, 4 H, C_6_H_4_), 7.32 (t, *J* = 7.9 Hz, 2 H, C_6_H_4_) ppm. ^13^C NMR (CDCl_3_, 125 MHz, 298 K): δ 193.9, 144.4, 134.7, 134.1, 129.1, 124.3, 120.3 ppm.

22. 3,4-dihydronaphthalen-1(2H)-one

^1^H NMR (CDCl_3_, 500 MHz, 298 K): δ 8.04 (d, *J* = 9.5 Hz, 1 H, C_6_H_4_), 7.46 (t, *J* = 9.5 Hz, 1 H, C_6_H_4_), 7.30 (t, *J* = 9 Hz, 1 H, C_6_H_4_), 7.25 (d, *J* = 9.5 Hz, 1 H, C_6_H_4_), 2.96 (t, *J* = 7.5 Hz, 2 H, CH_2_), 2.65 (t, *J* = 7.5 Hz, 2 H, CH_2_), 2.16-2.01 (m, 2 H, CH_2_). ^13^C NMR (CDCl_3_, 125 MHz, 298 K): δ 198.5, 144.6, 133.6, 132.6, 128.9, 127.2, 126.8, 36.2, 29.8 ppm.

23. 2, 3-dihydro-1H-inden-1-one

^1^H NMR (CDCl_3_, 500 MHz, 298 K): δ 7.75 (d, *J* = 7.7 Hz, 1 H, C_6_H_4_), 7.58 (t, *J* = 7.4 Hz, 1 H, C_6_H_4_), 7.48 (d, *J* = 7.7 Hz, 1 H, C_6_H_4_), 7.36 (t, *J* = 7.4 Hz, 1 H, C_6_H_4_), 3.16-3.13 (m, 2 H, CH_2_), 2.70-2.67 (m, 2 H, CH_2_) ppm. ^13^C NMR (CDCl_3_, 125 MHz, 298 K): δ 207.1, 155.2, 137.1, 134.6, 127.3, 126.7, 123.7, 36.2, 25.8 ppm.
